# A bolder conservation future for Indonesia by prioritising biodiversity, carbon and unique ecosystems in Sulawesi

**DOI:** 10.1038/s41598-022-21536-2

**Published:** 2023-01-16

**Authors:** Wulan Pusparini, Andi Cahyana, Hedley S. Grantham, Sean Maxwell, Carolina Soto-Navarro, David W. Macdonald

**Affiliations:** 1 Wildlife Conservation Research Unit (WildCRU), Department of Biology, Recanati-Kaplan Centre, University of Oxford, Oxford, UK; 2 Yayasan Konservasi Ekosistem Alam Lestari, 16610, Bogor, Indonesia; 3 Centre for Ecosystem Science, UNSW Australia, Sydney, NSW 2052 Australia; 4 School of Earth and Environmental Sciences, University of Queensland, Brisbane, Australia; 5 UN Environment Programme World Conservation Monitoring Centre, Cambridge, UK

**Keywords:** Ecology, Biodiversity, Conservation biology, Ecosystem ecology, Restoration ecology, Tropical ecology, Ecology, Biodiversity, Conservation biology, Tropical ecology

## Abstract

As more ambitious protected area (PA) targets for the post-2020 global biodiversity framework are set beyond Aichi Target 11, renew thinking into spatial prioritisation is required to enable PA expansion that maximises environmental values. Our study focuses on the biodiverse and forest-rich Indonesian island of Sulawesi, which has a terrestrial PA network that covers 10% of the island. We used Marxan to investigate trade-offs in the design of an expanded PA network that prioritised different conservation features (biodiversity, forest cover, carbon stock, karst and valuable metal-rich areas) under varying island-wide coverage targets (17%, 30%, and 50%). Our first scenario, which required existing PAs to be selected, required larger areas to meet these coverage targets, in contrast to our second scenario, which allowed for any part of the island to be chosen, irrespective of PA status. The vast Mekongga and Bangkiriang Landscapes, and Gorontalo corridor were consistently identified as a high priority for protection under all scenarios. To meet our conservation targets through expanding current PAs, creating new PAs, and creating corridors that connect existing PAs, we used a spatially explicit three-phase approach. Our findings identified 26,508 km^2^ of priority areas to be included in the current PA network, potentially assisting Indonesia in meeting its post-2020 GBF target, if our approach is replicated across Indonesia as a national or sub-national analysis. We discuss various land management options through other effective area-based conservation measures (OECMs) and the costs to deliver this strategy.

## Introduction

Protected areas (PAs) are a mainstay nature conservation strategy^1^. While they may not be a perfect solution^2–4^, PAs often represent the last strongholds for threatened species and intact habitats, and provide a buffer against environmentally damaging development^5–8^. Terrestrial PA targets are typically set as a percentage of total land area, despite the fact that focusing on quantitative aspects while ignoring qualitative aspects and robust indicators may fail to fully recognise differences in biodiversity richness and habitat types^9,10^. Nevertheless, over the past 10 years, most countries have been working towards the Convention on Biodiversity (CBD) Aichi targets, in particular Target 11, which calls for the protection of at least 17% of terrestrial and inland water areas^11^. A forthcoming post-2020 Global Biodiversity Framework (GBF) will likely include a revised PA target of 30% of land conserved by 2030^12–14^ and an ambitious target of 50% by 2050^15,16^. This raises the question of how and where this can be achieved and how it can be done in a way that complements the current PA network in each country.

As a signatory to the CBD, the Government of Indonesia is working to achieve the various Aichi targets and may further commit to the new post-2020 GBF 30% target. However, with 21.3% of its land area already under some form of protection, Indonesia has surpassed the Aichi Target 11 for terrestrial protection^17,18^. The Government of Indonesia is currently formulating a new set of national PA targets as part of its efforts to update its National Biodiversity Strategy and Action Plan 2015–2020 in response to the post-2020 GBF. Setting meaningful targets for 2030 requires increasing the representativeness of PAs in Indonesia and their management effectiveness, rather than simply expanding the network by only using an area-based approach. Here, we provide an Indonesian case study, using systematic conservation planning to explore how best to achieve the post-2020 GBF target by increasing the coverage and representativeness of important conservation features inside Sulawesi’s PA network.

Sulawesi is the eleventh largest island in the world. It is renowned for its biogeographical importance ever since Alfred Russel Wallace first explored it in the eighteenth century^19^. It is part of the Wallacea biodiversity hotspot^20,21^ and one of the 200 global ecoregions for conservation^22^. Despite its proximity to the islands of Borneo and Java, Sulawesi's biodiversity is unique^23–27^, with 98% of its non-flying mammal species, 37% of its bird species, and 26% of its reptile species being endemic^20,28–30^. Its extensive forests and mangroves store and sequester globally significant volumes of carbon^31,32^. The terrestrial area of Sulawesi spans 186,404 km^2^, of which 18,822 km^2^ (10.1%) is assigned within the island’s PA network. Of the 79 PAs in Sulawesi, Lore Lindu National Park (NP) was the first to be designated (in 1982) and Gandang Dewata NP the most recently created (2016), which indicates the government’s willingness, commitment, and ambition to expand its PA network. Sulawesi also has the world's largest nickel deposit^33^, which is currently the most sought-after element for the development of electric vehicle batteries^34^.

In this study, we conduct an island-wide spatial priority setting analysis for Sulawesi to inform PA planning and, more broadly, land-use management decision-making. A recent study^35^ identified synergies between carbon and biodiversity conservation planning at a broader scale across Asia, including Sulawesi. Cannon et al. (2007) carried out a similar study in Sulawesi, which was restricted to forest condition (old growth, good, fair, poor, and converted) and a data set from more than 20 years ago. This foundational study showed that the priority conservation areas for that period were consistent with the areas chosen by government and non-governmental organisations, with several additional priority areas identified outside of the PA system. Our analysis builds on these two studies by incorporating more recent and detailed data sets and using a spatial approach that, in addition to forest condition, considers other critical ecosystem types, biodiversity, and carbon stocks, with detailed data layers at a finer resolution, that enables site-specific management recommendations to be made. In contrast to the Key Biodiversity Areas (KBA), which only uses biodiversity data in its prioritization, spatial conservation planning can combine biodiversity and other conservation features that are relevant for species and habitat management^36^. Our study aimed to identify priority areas by setting targets under different conservation planning scenarios by: (1) performing a PA gap analysis; (2) selecting high-priority areas in addition to the current PA network; and, (3) identifying implementation opportunities and strategies for forest conservation across Sulawesi.

## Materials and methods

We used Marxan version 2.43 simulated annealing optimisation, with an iterative improvement method^37,38^, to enable systematic conservation planning for Sulawesi. Our goal was to determine how to prioritise potential new conservation areas when faced with multiple candidate sites (termed Planning Units and referred to as PUs, hereafter). We used a hexagonal grid of 1.3 km^2^ that served as the basic PUs within the landscape.

### Study area

The human population of Sulawesi in 2020 was 19.9 million people^39^. Two provinces in Sulawesi rank fifth (Gorontalo) and ninth (Central Sulawesi) highest for poverty amongst Indonesia’s 34 provinces^40^. In Indonesia and Sulawesi, there are 13 different types of conservation areas (Table S1) that are under different management authorities. The Ministry of Environment and Forestry (MoEF) is responsible for National Parks, Nature, Wildlife and Game Reserves, Nature Recreation Parks, and Hunting Parks, whereas Forest Management Units (such as Grand Forest Parks), whilst still the responsibility of MoEF, have devolved management to provincial government agencies.

The Wallacea biogeographic region, which includes Sulawesi, lost 10,231 km^2^ of forest between 2000 and 2018^41^ and 373 km^2^ of forest in 2017–2018^42^. These losses are unevenly distributed across the island, with the highest rates having occurred in the west and southeast lowland parts^43^. The threat of forest loss in Sulawesi, particularly in Central Sulawesi, is largely attributed to the occurrence of anthropogenic fires and past deforestation^41^. A more recent threat to conservation areas is legal and illegal mining, especially for nickel^44^. Most of this deforestation, and predicted future deforestation, occurs outside of the PA network^41^. To effectively mitigate these threats and protect Sulawesi's species and ecosystems, an island-wide spatial prioritisation approach is urgently needed.

### Conservation scenarios and priority setting objectives

Our goal was to identify potential PAs across Sulawesi. Our conservation values included forest cover, high carbon storage, distribution of endangered and critically endangered species, ultramafic soil, and karst. We included karst because it is a threatened ecosystem type, with limestone quarrying as the primary threat^45^ and high levels of species endemism^45–48^. Ultramafic soil is included because of its high plant endemicity^49,50^, and being rich in valuable, often-mined, metals such as nickel^51^.

The target was met by balancing with the estimated cost of protection in each PU. We investigated three scenarios for selecting priority conservation areas. The first scenario aimed to protect 17% of the identified conservation targets based on features such as carbon, karst, and forest cover. This goal is based on Aichi Target 11, which calls for the protection of 17% of terrestrial and inland water by 2020. The second scenario, which is based on Target 3 of the post-2020 Global Biodiversity Framework^52^, aimed to protect 30% of the identified conservation targets. The third scenario, based on the ‘Half-Earth concept’, sets the most ambitious target because it aimed to protect 50% of the identified conservation targets in Sulawesi^15,16,53^.

#### Conservation features

We used eleven conservation features and set targets based on the proportion of each feature under the three management scenarios (Table S2, Fig. S1). We classified conservation features into five categories: (i) carbon stocks (i.e., above and below ground, ABG, terrestrial carbon and soil carbon), using a data layer developed by^54^ to identify areas with high carbon stocks; (ii) forest types defined from the government’s official Land Use Land Planning 2018 dataset (MoEF 2019); (iii) karst (MoEF 2019); (iv) distribution of important animal species (IUCN Red List website); and, (v) ultramafic outcrops (MoEF 2019).

We combined primary dryland and secondary forest into a single forest cover layer and reclassified this based on their elevation range to include lowland (0–150 m asl), low elevation hill (150–500 m asl), medium elevation hill (500–900 m asl), sub-montane (900–1400 m asl), lower montane (1400–1900 m asl), montane (1900–2500 m asl), and tropical upper montane/sub-alpine (> 2500 m asl) (Laumonier, 1997).

We obtained species distribution maps from the IUCN Red List website (https://www.iucnredlist.org/search, accessed on March 16, 2022) using the following advance search category: Animalia is the Kingdom; Amphibia, Aves, Mammalia, and Reptilia are the Classes; CR and EN are the Red List Categories; and Sulawesi is the Land Region. We eliminated four species because they are not found in Sulawesi, possibly due to an error in the IUCN database (*Vanellus gregarious* and *Pterodroma baraui*), or because their distributions cover the marine area rather than the terrestrial (*Chelonia mydas* and *Eretmochelys imbricate*). We excluded VU species because several species, such as the Sulawesi giant squirrel, lacked fine-scale data and had distribution maps that were simply allocated to any forest patch on the island, rather than species occurrence. The final search yielded 12 mammals, 4 birds, 14 reptiles, and 3 amphibians (Table S3). The inhabitable area (non-forest area that has been developed or modified by humans) was removed from the spatial data composite of these 33 species distributions.

#### Ecological condition

We created a cost layer by combining land use and land cover with the ‘Forest Landscape Integrity Index’ developed by Grantham et al. (2020; Table S4). Cost layer refers to the loss or cost incurred by an area should it be assigned as a PA. No standard approach exists for determining the amount of penalty and cost, so we assigned a score to each PU that was proportional to how much of the area had been modified, less naturally intact or degraded. For example, the land use and land cover data contained 22 distinct categories designated by the Government of Indonesia^55^. We ranked them according to their natural habitat state: with natural habitats, such as primary and secondary forests, assigned a cost score of ‘1’, highly modified habitats, such as plantations and farming areas, assigned a score of ‘10’, and shrubs assigned a score of ‘5’. Eight of the 22 classes were either heavily developed or not a terrestrial system and were therefore left out of the analysis (i.e., settlement, bare land, water bodies, airport and seaport, transmigration area, mining concession, fishpond, and paddy field). The Forest Landscape Integrity Index combines observed and inferred forest pressure and fragmentation data to produce a continuous index ranging from 1 to 10, indicating the degree of anthropogenic modification^56^. We inverted the value of intact forests so that the most integrated had the lowest cost. We created a single cost layer from multiple factors^57^ by averaging the 15 cost factors (Table S4) in each PU, yielding a single cost value for each PU (Fig. S1).

### Priority area and gap analyses

**Figure 1:**
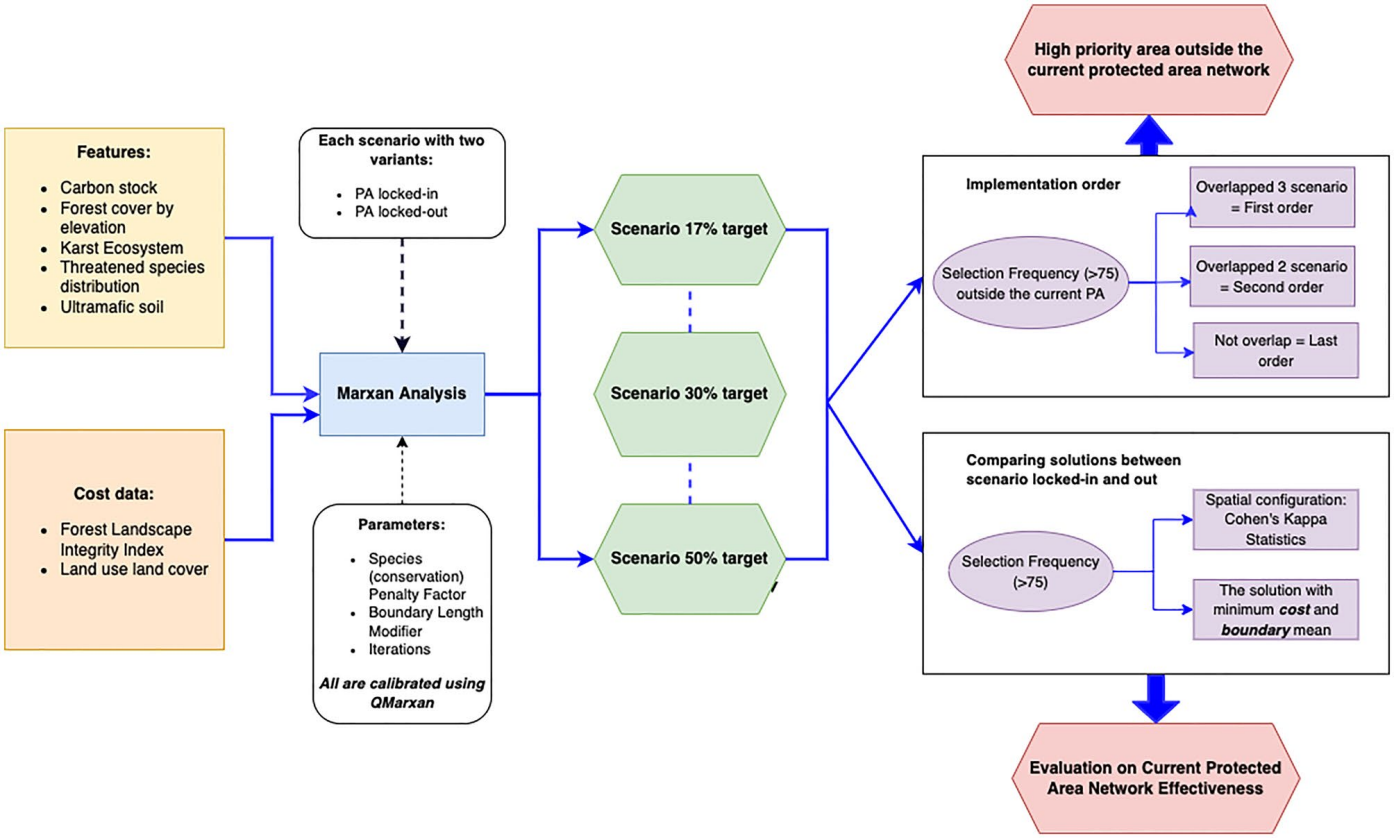
Workflow for identifying priority conservation areas in Sulawesi under three protection scenarios.

To meet the conservation features targets, ensure connectivity between areas, and reduce the total cost of priority area management (Fig. 1), we used Marxan v.4.0.6 to identify priority conservation zones through QMarxan Toolbox (2.0.1.), a QGIS 3.18.3 plugin (https://qgis.org/en/site/). We defined a total of 140,906 PUs on mainland Sulawesi and its outer islands, with each PU being a 1.3 km^2^ hexagon. Multi-edged PUs, such as hexagons, are more efficient in creating priority areas because they have a lower edge:area ratio than square grid cells. We ran the analysis in QMarxan for each scenario, starting with a Species Penalty Factor (SPF) of 1 and a constant boundary cost (1). The connectivity cost, also known as the boundary cost, is a value that represents connectivity; the higher the PU value, the more likely that it will be connected. By starting the analysis with a constant value, the algorithm ensures that all PUs are treated equally. We then calibrated the SPF and the Boundary Length Modifier (BLM). The SPF is calibrated to ensure that the penalties for missing conservation features are appropriately scaled and relative to one another. While the BLM is calibrated to find the best value that strikes a balance between area compactness and cost (as the value of the BLM increases, the algorithm will prefer a 'single large' design over a 'several small' designs, or increase 'connectivity'). To compare the spatial arrangement within and between scenarios, we used the kappa statistic^58^.

### Representativeness of Sulawesi’s protected area network

We ran each of the three protection scenarios in two ways: with a requirement that all existing PAs be included (scenario ‘a’); and, without such a requirement (scenario ‘b’). PAs were included to identify new priority areas outside of those already protected. In scenario ‘b’, PAs were excluded to allow for spatial assessments based on representativeness, allowing for comparisons with existing PAs to assess their efficacy in meeting the conservation targets^57^. We included 79 PAs (Table S1) from the World Database on PAs^59^. We ensured these were accurate and aligned with the official MoEF PA list. We based our recommendations to increase current PA coverage in Sulawesi on scenario ‘a’, which required all PAs to be included in the selection procedure. Nonetheless, because scenario ‘b’ is not biased towards the existing PAs, it is useful for highlighting gaps between the current PA network and the identified priority areas. If both scenarios select the same area, the weight is greater than if only scenario ‘a’ selects the area. We compared spatial similarities between scenarios using weighted kappa (κ) statistics, where 0 denoted no agreement, 0.01–0.20 low agreement, 0.21–0.40 fair agreement, 0.41–0.6 moderate agreement, 0.61–0.80 substantial agreement, and 0.81–1.00 the highest agreement^60^.

### Identifying high-priority areas outside the current protected area network

Marxan works by continually repeating the analysis to identify PUs that best achieve objectives. Due to the nature of the algorithm often each run can be slightly different. Selection frequency is the sum of how many times a PU was selected across all the runs. Areas designated as a priority have a high degree of selection frequency of more than 75 out of every 100 iterations. We merged the priority areas from the three percentage area scenarios and devised a plan for sequential implementation to make meeting this goal more realistic. We began by identifying high priority areas outside of the current PA network, as determined by the 17% scenario, and then expanded on this for the 30% and then 50% scenarios.

**Figure 2:**
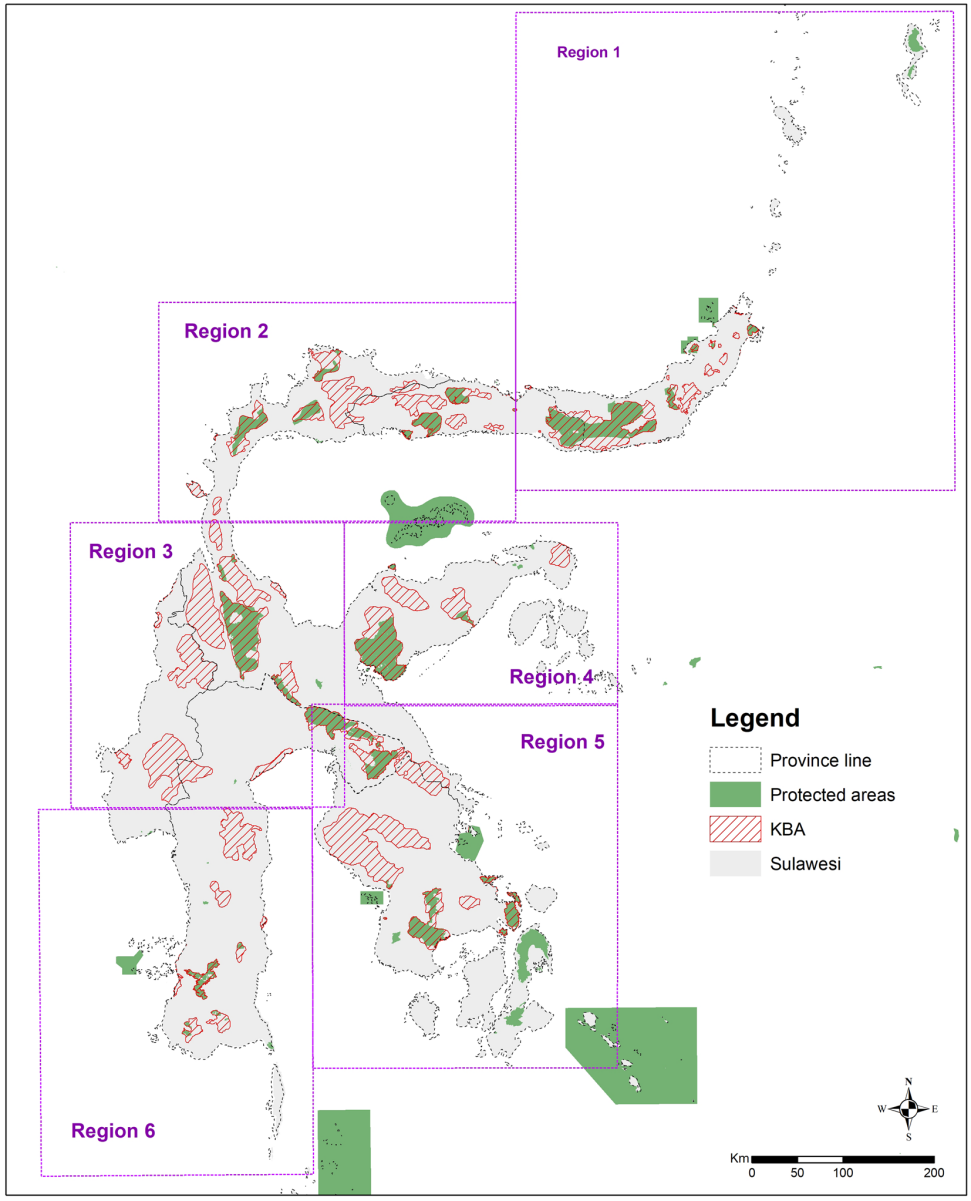
Geographic regions we used to define and identify new priority areas in Sulawesi.

Based on provincial delineation and governance structures, we divided the study area into six geographical regions (Fig. 2): (1) North Sulawesi Province; (2) Gorontalo and Central Sulawesi Provinces, covering Nantu Wildlife Reserve (WR) in the east and Gunung Sojol in the west; (3) Central and West Sulawesi Provinces, extending from Pegunungan Tokalekaju in the north and connecting Lore Lindu NP and Gandang Dewata NP as a large landscape, and Faruhumpenai Nature Reserve (NR) in the east; (4) Central Sulawesi Province's eastern region, which includes Balantak in the east, Gunung Lumut and Bangkiriang in the centre, and Morowali in the west; (5) Southeast Sulawesi Province, which includes the Routa KBA and Mekongga landscape in the north, and Buton Island in the south; and, (6) South Sulawesi Province.

The identified priority areas were overlaid on the KBAs^61^ to evaluate them in the context of landscape conservation in Sulawesi. We overlaid the identified priority areas with current active concessions to assess the potential threats of establishing the priority areas as conservation areas. Priority areas that fall within the scope of a concession are regarded as being under threat of clearance. Using MoEF data, we identified plantation concessions: (1) Natural Forest Management Permit (*Hak Pengusahaan Hutan*); (2) Palm Oil Concession; and, (3) Industrial Plantation Forest (*Hutan Tanaman Industri*). The mining concessions are public data made available by the Directorate General of Mineral and Coal, Ministry of Energy and Mineral Resources.

## Results

**Figure 3:**
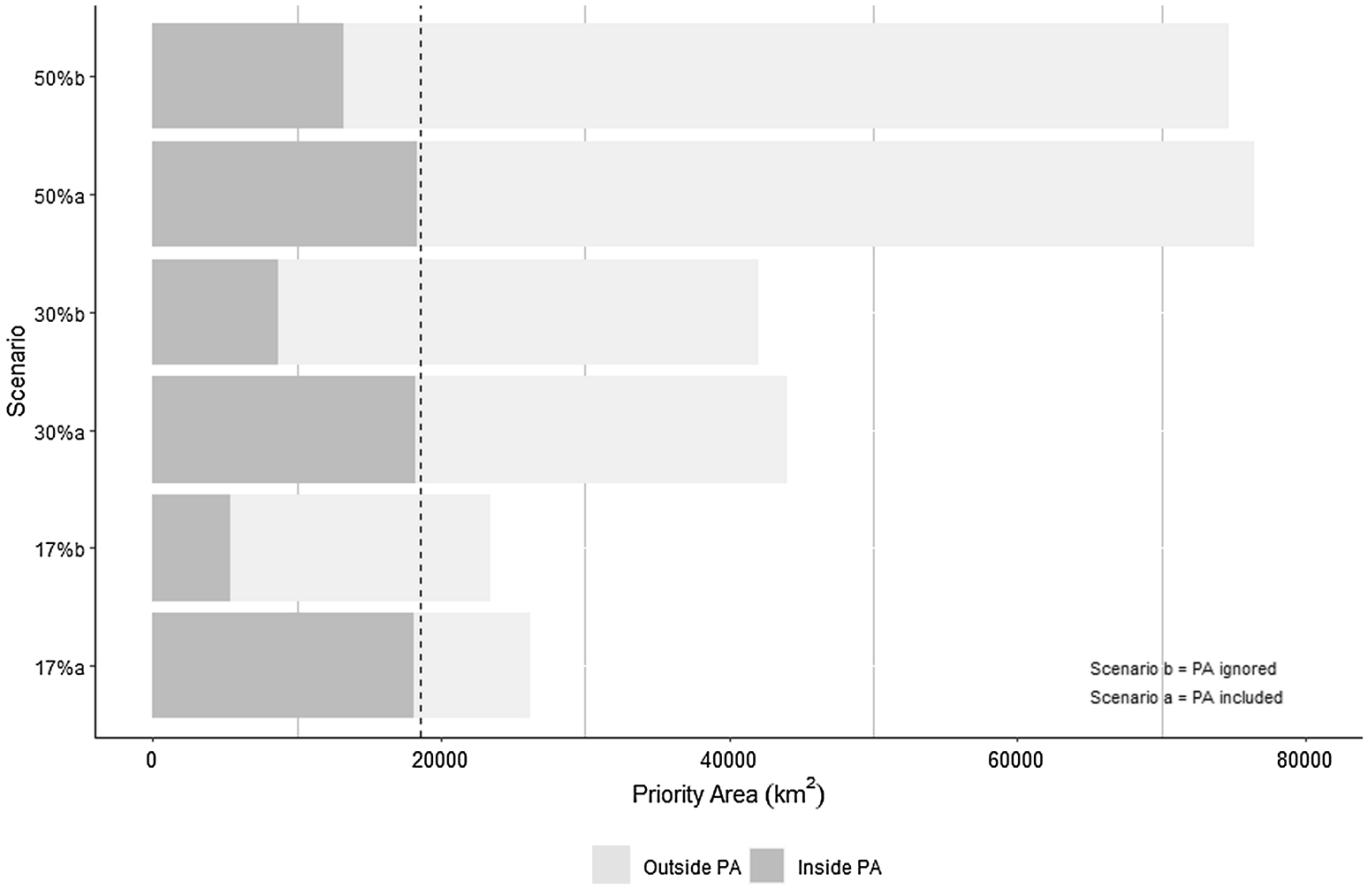
Sulawesi priority area size under six scenarios (two scenarios, ‘a’ and ‘b’, for protected area selection and three protection targets of 17%, 30%, and 50%). The dashed line denotes the total size of Sulawesi's current protected area network.

Given the same iterations, all percentage coverage targets under scenario 'b' (PAs are not required to be included) had a lower mean cost and were more compact with less edge. With the exception of the 17% target, they also had lower mean boundary lengths than those under scenario 'a' (PAs must always be included) (Table S5). Under scenario 'a' the total size of the priority areas was slightly larger than scenario ‘b’. When only those from outside the current PA were considered, the selections under scenario 'b' were always larger in size (Table 1). Scenario ‘b’ revealed a disparity between what we have and what should be. Scenario ‘b’ revealed gaps in the PA network by selecting a larger expanse of priority areas outside of this network than under scenario ‘a’, which was required to include these PAs. Scenario ‘b’ met the 17%, 30% and 50% targets through selecting a smaller number of PUs (Fig. 3).

**Table 1:** Size of the priority area (for planning units with a selection frequency > 75) in scenarios ‘a’ and ‘b’.

Scenario target	Scenario ‘a’		Scenario ‘b’	
	Total priority area (km^2^)	Priority area outside current PA (km^2^)	Total priority area (km^2^)	Priority area outside current PA (km^2^)
17%	26,162	8,007	23,480	18,058
30%	44,008	25,829	42,018	33,358
50%	76,398	58,019	74,626	61,430

Figure 3 shows that the size of all scenarios ‘a’ inside the PA are equivalent to the current PA size (the dashed line). While in scenario ‘b’, the size of priority areas inside PAs for all targets is less than the total area of the PAs. Due to the small size of the total priority area within PA in scenario 'b', several of the existing PAs were partially chosen or not chosen at all.

Within the same target, the weighted Kappa statistic between scenarios 'a' and 'b' describes the spatial congruence of the two scenarios. The weighted Kappa statistic was the lowest for the 17% target (κ = 0.30), moderate for the 30% target (κ = 0.58), and significant for the 50% target (κ = 0.75). The lower the percentage target would result in the algorithm prioritizing noticeably different areas to meet the target, since the current PAs did not adequately represent the five conservation features (forest, carbon, karst, species, and ultramafic soil). As the percentage targets increased, there was increasingly closer alignment between scenarios ‘a’ and ‘b’ for the size of the priority areas required to be selected to meet those targets.

**Figure 4:**
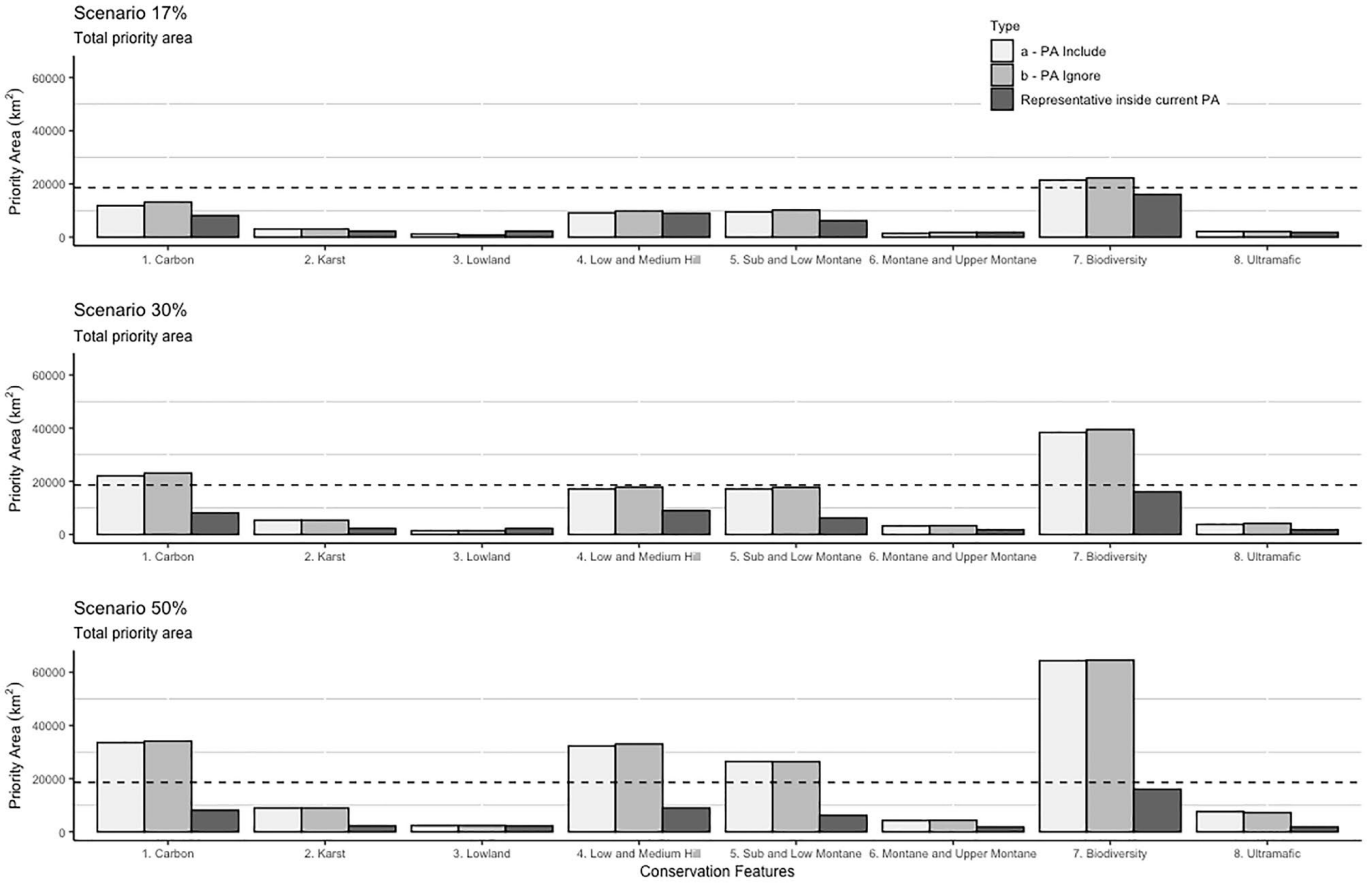
Sulawesi conservation features representativeness for all target scenarios (17%, 30%, and 50% protection target). The dashed line denotes the current protected area network on Sulawesi (18,602.4 km^2^).

Sulawesi's network of 79 PAs currently covers 16% of the island’s 2018 forest area (15,375.3 km^2^), 9% of its karst (2,260.9 km^2^), 15% of the distribution of important species (16,023.4 km^2^), and 12% of its ultramafic soil (15,362.9 km^2^). Only 18% (8,109 km^2^) of the island's top quantile for soil carbon organic is located in its PA network. Including these priority areas in the current PA network increases the representation of conservation features (Fig. 4). Even the modest target of 17% increases area representativeness for important species (biodiversity), carbon, and the sub montane – lower montane forest. The more ambitious targets of 30% and 50% increase representation of all conservation features, apart from lowland forest, which is almost entirely included in the current PA network.

The size of the priority areas identified outside of the current PA network is 58,483 km^2^ in scenario ‘a’ and 62,458 km^2^ in scenario ‘b’ (Table 2). These areas are vast and would require more than three times the current land area that is presently assigned for PAs. To achieve this expansion goal, we propose a three-stage implementation plan based on the overlap (i.e. order of importance) amongst the three percentage target scenarios under ‘a’ and ‘b’.

**Table 2:** Recommended order for assigning protection to priority areas outside of Sulawesi's current protected area network.

Order	Scenario ‘a’ (km^2^)	Scenario ‘b’ (km^2^)
First	6,864	14,911
Second	19,644	20,565
Third	31,975	26,982
Total area	58,483	62,458

**Figure 5:**
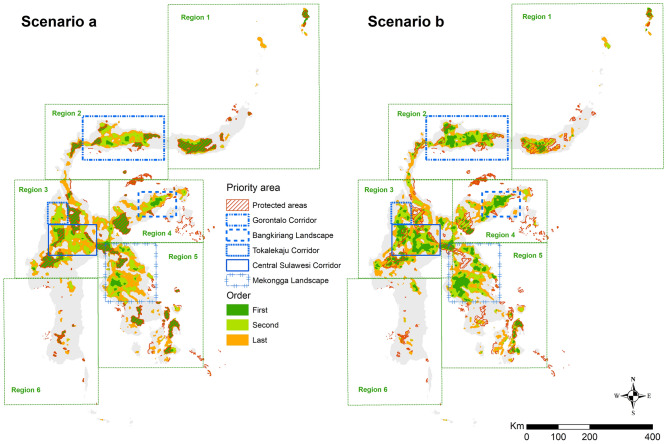
Priority areas in Sulawesi based on scenarios ‘a’ (in which the current PA network had to be included in the selection) and ‘b’ (in which any area could be selected), as well as the proposed order of selection for protecting these areas.

Our first recommended implementation order prioritises 6,864 km^2^ under scenario ‘a’ (Table 2, Fig. 5) with 35% of this area being located within three large clusters that are not connected to the current PA network. The largest areas is the Mekongga landscape (Region 5) with the single largest first order priority site (609.4 km^2^) being outside of the PA, followed by the 580 km^2^ Bangkiriang landscape (Region 4), and 892.6 km^2^ Gorontalo corridor (Region 2) (Fig. 5).

A compilation of areas covering 19,644 km^2^ is prioritized in the second order under scenario ‘a’ (Table 2). These areas are significantly larger and more extensive than the current PA network. To make achieving this goal more feasible, we propose prioritizing five clusters. The first three are those mentioned above in the first order description, with the remaining two being the Tokalekaju landscape and the Central Sulawesi corridor. Under the second order, the Mekongga landscape (consisting of three patches) extends further to cover a 3346.9 km^2^ which combines with the first order area, and additionally the 1652.1 km^2^ Bangkiriang landscape with the first order area as its core. The extensive Gorontalo corridor (Popayato-Paguat-Buol-Tolitoli-Dako Mountain) spans 4729.6 km^2^, with the first order area serving as the core (892.6 km^2^). The Tokalekaju Mountain landscape (1145.4 km^2^) is a large single second order area (with 63 km^2^ of the first order), and the central Sulawesi corridor (2751.1 km^2^) that connects Gandang Dewata NP, Lore Lindu NP, and Pamona NR (KBA Danau Poso. The second order under scenario ‘a’ aligns with Target 3 of the first draft of the post-2020 GBF by: adding a buffer area to the existing PAs; creating new standalone PAs; and, creating corridors that connect pre-existing PAs.

Focusing on the first and second order priorities of scenario 'a,' which together create 26,508 km^2^ priority areas that are additional to the current PA network, we describe these results in greater detail for each region, considering comparisons to scenario 'b' and potential impacts from different land management sectors.

### North Sulawesi (Region 1)

Aside from the PAs, the first order scenario 'a' adds 1,121.4 km^2^ to the low and medium hill forest border of Bogani Nani Wartabone NP and Karakelang WR (420.1 km^2^) (Fig. S2). In comparison, scenario 'b' emphasizes the significance of KBA Gunung Simbalang, with a third order priority area that is larger than the KBA itself and a first and second order priority area at its centre. Scenario 'b' highlights the significance of Sangihe Island as a second order priority rather than the last order in scenario 'a' (Fig. S3). The PT Tambang Mas Sangihe concession, which covers half of Sangihe Island (https://geoportal.esdm.go.id/minerba/), is located in the southern extent of this area. Another potential threat is the priority area along the northern boundary of Bogani Nani Wartabone NP, which overlaps with the PT Huma Sulut Natural Forest Management Permit area, suggesting that it could be logged in the future. This region’s karst is located within the Karakelang landscape, outside a PA.

### Gorontalo and Central Sulawesi (Region 2)

Outside of the PAs, the first and second order priority areas of scenario ‘a’ includes 4,729.6 km^2^ that overlaps with KBA Popayato-Paguat (first order priority area of 461.4 km^2^) and KBA Buol-Tolitoli (first order priority area of 317.4 km^2^) which we term as the Gorontalo corridor, dominated (68%) by low and medium elevation hill forest. The first order priority area in this landscape extends the border of Nantu NR, Gunung Dako NR, and Tinombala NR (Fig. S2). Outside of the PA network, this region contains a large portion of the top quantile for carbon stock. This condition is illustrated by scenario ‘b’ in which the priority areas of the first and second order areas are located outside of the PAs and heavily overlap with the KBAs (Fig. S3), and which covers all of the high carbon areas. The Gorontalo corridor of scenario ‘b’ encircles the area, with an oil palm concession in the centre (251 km^2^). This region includes PT Sentral Pitulempa and PT Taman Hutan Asri’s logging concession areas (HPH), as well as PT Inti Global Laksana and PT Cipta Cakra Murdaya’s oil palm concessions. Furthermore, the mining concession of PT Gorontalo Sejahtera overlaps with the southern part of Panua NR (Fig. S2 and S3, code PA ID 15).

### Central Sulawesi (Region 3)

Outside of the PAs, this is the largest region and covers the most extensive priority areas. Based on scenario ‘a’, there are two stand-alone priority areas worth mentioning: the Tokalekaju landscape and the Central Sulawesi corridor (Fig. S2). The Tokalekaju landscape covers 1,146.7 km^2^ and is dominated by sub and low montane hill forest type. The Central Sulawesi corridor spans 3,645.5 km^2^ and is dominated by low montane forest. The first order priority of the Central Sulawesi Corridor (872 km^2^) is mostly an extended border of Lore Lindu NP, Gandang Dewata NP (PA ID 37), and a small polygon west of Pamona NP (PA ID 28). The Tokalekaju landscape, KBA Pambuang, and the Central Sulawesi corridor are connected as one large landscape in scenario ‘b’ with a significant amount of first order priority areas outside the current PAs (Fig. S3).

The overlap of Pegunungan Tokalekaju KBA with the Natural Forest Management Permit area of PT Satya Sena Indratama, Pambuang KBA with the Natural Forest Management Permit area of PT Inhutani I Mamuju, and Sulawesi Tengah Grand Forest Park with the mining concession PT Citra Palu Mineral indicate the potential development impacts that may occur in this region. Some 40% of the Tokalekaju Landscape is under concessions (456.4 km^2^) belonging to three companies with National Forest Management Permits. One of them, PT Satya Sena, has a concession that overlaps with 15.6 km^2^ of a first order priority area. Furthermore, 560.4 km^2^ of the Central Sulawesi corridor is within a concession, primarily the Natural Forest Management Permits of PT Rante Mario and PT Inhutani I Mamuju.

### Eastern Sulawesi (Region 4)

Under scenario ‘a’, the Bangkiriang landscape is the only large standalone polygon of first and second priority (1,678.8 km^2^) (Fig. S2). It stretches from Lombuyan I and II WR (PA ID 35) to Bangkiriang WR (PA ID 33), with a first priority area of 602.7 km^2^ at its core (Fig. S2). The Bangkiriang landscape is dominated by submontane forest (44%, 750.9 km^2^), contains a significant amount of karst (56%, 934.3 km^2^), and because it is part of the East Sulawesi crescent geology area, it has a significant amount of ultramafic soil (32%, 531.4 km^2^). Scenario ‘b’ shows the importance of priority areas outside of PAs by selecting the majority of first and second priority areas outside of the PA network (Fig. S3), with a smaller first priority area inside Morowali NR (PA ID 32).

Concessions with Natural Forest Management Permits (37%, 631.2 km^2^), including PT. Bina Balantak Raya, PT. Dahatama Adi Karya, PT. Palopo Timber Company, and PT. Satyaguna Sulajaya, may have forest impacts. The first company is the largest, with 292 km^2^ of its concession overlapping with the KBA Bangkiriang. Another potential impact is from the PT Sinar Makmur Cemerlang’s mining concession that is located within the KBA Bangkiriang.

### Southeast Sulawesi (Region 5)

Despite having a dense forest cover and being part of the East Crescent Sulawesi geology, this region has very few PAs. There is no PA between Danau Towuti NRP (PA ID 45) and Rawa Aopa Watumohai NP (PA ID 65). Based on our scenarios ‘a’ and ‘b’, this gap in area is prioritized. Scenario ‘a’ identified three large standalone patches of first and second priority areas that overlap the KBA Mekongga and cover 3,346.9 km^2^ (Fig. S2). The largest patch, with an area of 2,406.8 km^2^, is dominated by submontane forest (first order area of 874.7 km^2^). This patch contains karst (854.7 km^2^) and Mekongga’s only patch of ultramafic soil (301.7 km^2^). Scenario ‘b’ depicts these three patches as a single continuous landscape with a large first order and a second order in the buffer zone (Fig. S3).

The second important standalone first and second priority area is 828.0 km^2^ of forest that overlaps with the KBA Routa (Fig. S2). The Routa landscape is dominated by a medium elevation hill forest (460.1 km^2^) and contains both karst (165.3 km^2^) and ultramafic soil (385.5 km^2^). The final significant standalone area is an unnamed landscape located east of Danau Matano (PA ID 43) and Danau Mahalona (PA ID 44) (Fig. S2). This is a 740.0 km^2^ area dominated by medium-elevation hills (368.8 km^2^) and a significant proportion of ultramafic soil (63%, 468 km^2^). The Routa landscape and the unnamed landscape to the north are depicted as one continuous landscape in scenario ‘b’ (Fig. S3). There are no identified threats from concessions in this region.

### South Sulawesi (Region 6)

This cluster has the least forest cover, the fewest PAs, and the fewest priority areas outside of the PAs. According to scenario ‘a’, the only large standalone area are the second and third order priority areas that overlap with the KBA Pegunungan Latimojong, which contains Sulawesi’s highest mountain. The second order priority area is in the landscape’s centre (222.7 km^2^), and contains 83% of montane and upper montane forest (Fig. S2). Scenario ‘b’ emphasizes the significance of the Latimojong landscape by identifying it as a first-order priority (Fig. S3).

## Discussion

The Sulawesi terrestrial PA network covers 10% of the island’s land surface. Our study provides an innovative approach to systematically enhance this network through three sequential stages, by incorporating new areas based on their key environmental attributes. Our results reveal that the current PA system protects less than 20% of the areas identified by our analysis as being priorities for the five conservation features (Table S2). The identification of 26,508 km^2^ (first and second order areas combined, Table 2) under the 30% target outside of the current PA network exemplifies this lack of representation. Assuming that all signatory Parties will adopt the CBD’s 30% target, addressing the funding gap to achieve this is critical, yet challenging. Obtaining the first order priority areas would increase PA coverage by 14% of the island and adding the second order priority areas would increase this to 24%. Adding all the areas identified from first to third order would cover 42% of the island’s land area.

Mackinnon and Mackinnon (1986) evaluated PA effectiveness in the Indo-Malayan Realm in the 1980s. Except for Dumoga-Bone NP (now Bogani Nani Wartabone NP), Tangkoko Dua Saudara NR, and Lore Lindu NP, they concluded that Sulawesi's PAs were generally inadequately protected. Our work reveals a shifting baseline with regards to the essential areas to protect. It also benefits from detailed data layers and analytical techniques that have recently become available. There are considerable gaps in the areas identified as priorities in the PAs exclusion scenario (‘b’) compared to those considered when Sulawesi’s PAs were established nearly 40 years ago. Three of the six PAs identified as essential by MacKinnon and MacKinnon, 1986 are not identified as priorities under scenario 'b' (Tangkoko Dua Saudara, Bantimurung, and Rawa Aopa). Nonetheless, both our research and that of MacKinnon and MacKinnon (1986) identify Lore Lindu NP and Bogani Nani Wartabone NP as conservation priorities.

Tangkoko-Dua Saudara is an important area due to its high conservation value. For example, it is home to a significant portion of the Critically Endangered black-crested macaque (*Macaca nigra*) population^62^. The *Macaca nigra* distribution was included in our analysis (Table S4), but the Tangkoko-Dua Saudara is still excluded under scenario 'b', possibly due to its small size and low irreplaceability in comparison to other larger patches. The omission of Bantimurung-Bulusaraung NP from our priority list may be explained by the Mekongga and Bangkiriang landscapes incorporating most of the karst, the primary feature of this park. Rawa Aopa NP has lost nearly half of its forest cover since it was gazetted in 1990, lowering its priority for selection and resulting in the selection of large landscapes such as Mekongga. The PA exclusion scenario is useful because it highlights important areas that are outside of the current PA network. We stress that our findings do not imply that the PAs not included in our analysis have no conservation value because they still serve many other important functions, such as for endemic species and ecosystem services.

There is considerable overlap between the critical areas highlighted in our study (Fig. 5) and KBAs, which further validates the prioritisation exercise conducted by the Critical Ecosystem Partnership Fund^61^. However, both the KBA and priority areas highlighted in our study are not included in the existing PA network. The same situation was encountered when we assessed connectivity as one crucial aspect of PAs. Many priority areas identified in this study were also part of the KBA corridor. For example, the KBAs of Bangkiriang and Mekongga were identified by our study as being part of the much larger Bangkiriang and Mekongga landscapes, respectively. The islands of Sangihe and Talaud, which were identified in this study as a priority and were also part of the CEPF priority corridor for North Sulawesi Province^61^. KBAs are based on species data, whereas our analysis extends beyond just biodiversity. One novelty in our approach is the inclusion of ultramafic soil, which alongside altitude often shapes the diversity of plants, the kingdom of species rarely considered in biodiversity prioritization exercises^50,63–65^. Our approach, de facto, would increase the focus on the many endemic plant species that continue to be described in Sulawesi^27,66^.

The priority areas identified in our analyses provide an opportunity to understand where conservation gains, in terms of coverage and ecological representativeness, could be made by expanding the PA network in Sulawesi. For example, to achieve the 17% protection target, the first order selection areas are located in the existing PA buffer zones. We propose that the most feasible approach to conserve these areas is a government-led PA enlargement and buffer management strategy. Once this has been achieved, the next step will be to protect the second order priority areas, mainly by designating a corridor system in region 2 (Gorontalo and Central Sulawesi). Many PAs in region 2 (Gunung Dako NR, Tinombala NR, and Gunung Sonjol NR) would benefit from increased protection status beyond their current designations.

For a continuous large landscape that is not connected to any PA, notably Mekongga and Bangkiriang, another type of protection is required. Parts of the two landscapes are also managed by the local government as part of Indonesia's watershed forest, also known as Protection Forest (*Hutan Lindung*). They typically have a lower protection status than national parks because there is no technical implementation unit, unlike national parks, that is directly responsible for them and, as a result, no direct funding for their protection. An ‘Essential Ecosystem Area’ is another plausible option, although their size is generally much smaller than that of a PA. For example, in 2017 the 0.15 km^2^ Taman Keanekaragaman Hayati (Biodiversity Park) Oheo Essential Ecosystem Area in North Konawe Regency^67^, was established near the Mekongga landscape. Given their size and importance, the Mekongga and Bangkiriang landscapes may even merit national park status and the increased resources that this would bring. This study supports Mekongga's previous effort to promote the area's status as a national park^68,69^.

The post-2020 GBF Target 3 aims to protect at least 30% of all land and sea, globally, and will likely be adopted by all CBD signatories^12,14^. Under the Mitigation and Conservation Hierarchy^70^, our work would contribute towards the first of four steps—‘Refrain’—by avoiding impacts to, and therefore retain, biodiversity. Our work identifies high priority areas to be considered for protection. To achieve the target of increasing the existing 10% area protected in Sulawesi to 17%, 30%, and 50%, the principles of equity and collaboration with neighbouring communities prioritised under various MoEF initiatives, such FOLU Net Sink 2030, should be followed.

One option would be through OECMs with long-term conservation effectiveness outside of the designated PA network^71^. A community-managed forests, such as Village Forest (*Hutan Desa*) schemes, offer an option for expanding the conservation area network^72,73^, as do privately managed restoration concessions, which could be managed under an Ecosystem Restoration Concession licence. Another framework such as indigenous lead conservation area could also be considered. Currently there 207,000 km^2^ of indigenous land in Indonesia, however only 15% of it are certified ^74^. In Sulawesi there are 16,000 km^2^ of indigenous land with the largest in the South Sulawesi province with 8,188.4 km^2^ ^75^. The second largest is in the Central Sulawesi province with 6,298.2 km^2^, this include five indigenous lands overlapping the Bangkiriang landscape: Taa Mpoa (168.5 km^2^), Taa Salaki (236.7 km^2^), Taa Vananga Bulang (220.1 km^2^), Lipu Wana Posangke (305.8 km^2^), and Wana Kayupoli (179 km^2^)^76^.

Increasing PA coverage is a key first step towards meeting the target of being nature positive by 2030 set by the Global Goal for Nature (www.naturepositive.org). Following the first ‘Retain’ step, the Mitigation and Conservation Hierarchy’s subsequent steps are to ‘Reduce’ the impact (e.g. through an agri-environment scheme), ‘Restore’ (e.g. ecosystem restoration concession), and ‘Renew’ (e.g. ecosystem creation or rewilding;^70^. Reducing the environmental impact of mining and plantation concessions is a top priority. This is particularly important for the conservation of many species, such as the critically endangered Cerulean paradise flycatcher (*Eutrichomyias rowleyi*) that is endemic to Mount Sahendaruman, Sangihe Island. This species is highly vulnerable to habitat change because its entire range is within the PT Tambang Mas Sangihe gold mining concession, which encompasses half of Sangihe island. The Sangihe Island’s significance was recognized as early as the early 80 s by^20^, who prioritised it for reserve establishment.

The mitigation hierarchy’s third step of restoring degraded areas aligns with the post-2020 target of restoring 20% of degraded terrestrial land and ensuring landscape connectivity. Indonesia may still fall short of this goal, with 7.6% (1,917 km^2^) of degraded land on Sulawesi being assigned for protection. The final mitigation hierarchy step of renewal, or rewilding, is still a foreign concept in Indonesia, but one that is gaining popularity^77,78^.

There is no set number for protection percentage that will guarantee that the PA network ensures the survival of ecosystem processes and biodiversity. However, in the case of relatively small, safeguarded areas, it appears safe to say that, in general, the higher the percentage target, the better. Decisions must be science-based while also considering equity for rural communities that rely on natural resources and benefit from healthy ecosystem services^79^. Although the focus of this study is on potential governance models, such as strictly managed PAs, to protect these vital but currently unprotected areas, it should be noted that protection does not always imply a fenced park. Other more participatory options, such as local community and indigenous people participation in conservation area management, are important and could be accommodate under future OECM designation, should this become an option. In the meantime, advancing Indonesia’s FOLU Net Sink 2030 strategy remains vital to safeguarding Sulawesi's rich natural capital while providing critical benefits to its people.

## Supplementary Information

Supplementary Information.

## Data Availability

All data used in this study is publicly available. The boundaries of protected areas can be found at https://www.protectedplanet.net/country/IDN. Sulawesi's Land Use, Land Cover, and Forest Cover and the geology – ultramafic soil layer can be found at https://tanahair.indonesia.go.id/portal-web/inageoportal/#/. The Forest Landscape Integrity Index can be found online at https://www.forestlandscapeintegrity.com/. Sulawesi's karst layer can be found at https://geoportal.esdm.go.id/. Carbon data can be obtained from Soto-Navarro et al. (2020). Concession maps can be found at: (i) industrial plantation forest - https://nusantara-atlas.org/, (ii) natural forest management permit - http://www.dephut.go.id/index.php/news/details/9671, (iii) palm oil - http://appgis.dephut.go.id/appgis/kml.aspx. The mining concession data are from https://geoportal.esdm.go.id/minerba/. The maps in this study, including those in the Supplementary Information, were created using GQIS Desktop 3.18.3 (https://qgis.org/en/site/). The priority area polygons resulting from this study are available at https://osf.io/gdtuy/?view_only=373163da22a04a26a9675d631a95930b.
