# Supplementary material for: A bolder conservation future for Indonesia by prioritising biodiversity, carbon and unique ecosystems in Sulawesi
Source: Sci Rep. 2023 Jan 16;13:842. doi: 10.1038/s41598-022-21536-2 (PMC9842766; doi:10.1038/s41598-022-21536-2)
Supplement: Supplementary file 1 — Supplementary Information. [file 41598_2022_21536_MOESM1_ESM.docx]

A Bolder Conservation Future for Indonesia by Prioritising Biodiversity, Carbon and Unique Ecosystems in Sulawesi

Wulan Pusparini^1*^, Andi Cahyana^2^, Hedley S. Grantham^3^, Sean Maxwell^4^, Carolina Soto-Navarro^5^, David W. Macdonald^1^

^1^Wildlife Conservation Research Unit (WildCRU), Department of Biology, Recanati-Kaplan Centre, University of Oxford, UK.

^2^ Yayasan Konservasi Ekosistem Alam Nusantara, Bogor, Indonesia.

^3^ Centre for Ecosystem Science, UNSW Australia, NSW 2052, Australia

^4^ University of Queensland, School of Earth and Environmental Sciences, Brisbane, Australia.

^5^ UN Environment Programme World Conservation Monitoring Centre, Cambridge, UK.

^6^Department of Biology, Fakultas Matematika dan Ilmu Pengetahuan Alam, Universitas Indonesia, Kampus UI, Depok, Indonesia.

*Corresponding author: [wulan.pusparini@lmh.ox.ac.uk](mailto:wulan.pusparini@lmh.ox.ac.uk)

# Supplementary Material

## Tables

**Table S1** – List of protected areas in Sulawesi

| ID | NAME | Designated (IND) | Designated (ENG) | Management Authority* | Area (Km^2^) |
| --- | --- | --- | --- | --- | --- |
| 1 | Karakelang | Suaka Margasatwa | Wildlife Reserve | BBKSDA Sulawesi Selatan | 286.30 |
| 2 | Batu Putih | Taman Wisata Alam | Nature Recreation Park | BBKSDA Sulawesi Selatan | 6.49 |
| 3 | Gunung Dua Saudara | Cagar Alam | Nature Reserve | BBKSDA Sulawesi Selatan | 72.27 |
| 4 | Batu Angus | Taman Wisata Alam | Nature Recreation Park | BBKSDA Sulawesi Selatan | 6.37 |
| 5 | Bunaken | Taman Nasional | Marine National Park | National Park Agency of Bunaken | 51.02 |
| 6 | Gunung Tumpa (Haveworang) | Taman Hutan Raya | Grand Forest Park | District Gov. of North Sulawesi Province | 2.09 |
| 7 | Gunung Lokon | Cagar Alam | Nature Reserve | BBKSDA Sulawesi Selatan | 7.76 |
| 8 | Gunung Manembo-nembo | Suaka Margasatwa | Wildlife Reserve | BBKSDA Sulawesi Selatan | 61.84 |
| 9 | Gunung Ambang | Cagar Alam | Nature Reserve | BBKSDA Sulawesi Selatan | 150.03 |
| 10 | Gunung Ambang | Taman Wisata Alam | Nature Recreation Park | BBKSDA Sulawesi Selatan | 25.95 |
| 11 | Bogani Nani Wartabone | Taman Nasional | National Park | National Park Agency of Bogani Nani Wartabone | 2,833.99 |
| 12 | Desa Olele (Bone Bolango) | Kawasan Konservasi Perairan Daerah | Locally Managed Marine Area | District/Local Government assisted by the Ministry of Marine Affairs & Fisheries (MMAF) | 0.03 |
| 13 | Tangale | Cagar Alam | Nature Reserve | BKSDA Sulawesi Utara | 1.01 |
| 14 | Nantu | Suaka Margasatwa | Wildlife Reserve | BKSDA Sulawesi Utara | 516.98 |
| 15 | Panua | Cagar Alam | Nature Reserve | BKSDA Sulawesi Utara | 365.34 |
| 16 | Kawasan Konservasi Perairan Daerah Boalemo | Kawasan Konservasi Perairan Daerah | Locally Managed Marine Area | District/Local Government assisted by the Ministry of Marine Affairs & Fisheries (MMAF) | 1.78 |
| 17 | Tanjung Panjang | Cagar Alam | Nature Reserve | BKSDA Sulawesi Utara | 30.42 |
| 18 | Pinjan/Tanjung Matop | Suaka Margasatwa | Wildlife Reserve | BKSDA Sulawesi Tengah | 17.58 |
| 19 | Gunung Dako | Cagar Alam | Nature Reserve | BKSDA Sulawesi Tengah | 197.28 |
| 20 | Tinombala | Cagar Alam | Nature Reserve | BKSDA Sulawesi Tengah | 359.63 |
| 21 | Gunung Sojol | Cagar Alam | Nature Reserve | BKSDA Sulawesi Tengah | 685.30 |
| 22 | Tanjung Santigi | Suaka Margasatwa | Wildlife Reserve | BKSDA Sulawesi Tengah | 17.21 |
| 23 | Teluk Tomini Kabupaten Parigi | Kawasan Konservasi Perairan Daerah | Locally Managed Marine Area | District/Local Government assisted by the Ministry of Marine Affairs & Fisheries (MMAF) | 27.26 |
| 24 | Pangi Binangga | Cagar Alam | Nature Reserve | BKSDA Sulawesi Tengah | 61.51 |
| 25 | Sulawesi Tengah | Taman Hutan Raya | Grand Forest Park | Pemerintah Daerah Provinsi Sulawesi Tengah | 73.24 |
| 26 | Wera | Taman Wisata Alam | Nature Recreation Park | BKSDA Sulawesi Tengah | 3.49 |
| 27 | Lore Lindu | Taman Nasional | National Park | National Park Agency of Lore Lindu | 2,153.57 |
| 28 | Pamona | Cagar Alam | Nature Reserve | BKSDA Sulawesi Tengah | 273.08 |
| 29 | Landusa Tomata | Taman Buru | Game Reserve | BKSDA Sulawesi Tengah | 40.48 |
| 30 | Kepulauan Togean | Taman Nasional | Marine National Park | National Park Agency of Kepulauan Togean | 304.03 |
| 31 | Tanjung Api | Cagar Alam | Nature Reserve | BKSDA Sulawesi Tengah | 31.82 |
| 32 | Morowali | Cagar Alam | Nature Reserve | BKSDA Sulawesi Tengah | 2,132.65 |
| 33 | Bangkiriang | Suaka Margasatwa | Wildlife Reserve | BKSDA Sulawesi Tengah | 122.81 |
| 34 | Kabupaten Banggai, Banggai Kepulauan, dan Banggai Laut | Taman Pesisir dan Taman Pulau Kecil | Coastal and Small Island Park | District/Local Government assisted by Ministry of Marine Affairs & Fisheries (MMAF) | 126.93 |
| 35 | Lombuyan I and II | Suaka Margasatwa | Wildlife Reserve | BKSDA Sulawesi Tengah | 29.66 |
| 36 | Pati-Pati | Suaka Margasatwa | Wildlife Reserve | BKSDA Sulawesi Tengah | 30.69 |
| 37 | Ganda Dewata | Taman Nasional | National Park | BBKSDA Sulawesi Selatan | 2,135.47 |
| 38 | Tahura Sulbar | Taman Hutan Raya | Grand Forest Park | Provincial Forestry Service | 9.37 |
| 39 | Kabupaten Polewali Mandar | Kawasan Konservasi Perairan Daerah | District Marine Protected Areas | Not Reported | 4.79 |
| 40 | Faruhumpenai | Cagar Alam | Nature Reserve | BBKSDA Sulawesi Selatan | 908.93 |
| 41 | Ponda-Ponda | Cagar Alam | Nature Reserve | BBKSDA Sulawesi Selatan | 0.81 |
| 42 | Kalaena | Cagar Alam | Nature Reserve | BBKSDA Sulawesi Selatan | 1.09 |
| 43 | Danau Matano | Taman Wisata Alam | Nature Recreation Park | BBKSDA Sulawesi Selatan | 232.18 |
| 44 | Danau Mahalona | Taman Wisata Alam | Nature Recreation Park | BBKSDA Sulawesi Selatan | 22.90 |
| 45 | Danau Towuti | Taman Wisata Alam | Nature Recreation Park | BBKSDA Sulawesi Selatan | 621.41 |
| 46 | Nanggala III | Taman Wisata Alam | Nature Recreation Park | BBKSDA Sulawesi Selatan | 9.66 |
| 47 | Sidrap | Taman Wisata Alam | Nature Recreation Park | BBKSDA Sulawesi Selatan | 2.84 |
| 48 | Lejja | Taman Wisata Alam | Nature Recreation Park | BBKSDA Sulawesi Selatan | 14.21 |
| 49 | Cani Sirenreng | Taman Wisata Alam | Nature Recreation Park | BBKSDA Sulawesi Selatan | 37.64 |
| 50 | Bantimurung Bulusaraung | National Park | National Park | National Park Agency of Bantimurung Bulusaraung | 434.27 |
| 51 | Kabupaten Pangkajene Kepulauan | Kawasan Konservasi Perairan Daerah | Locally Managed Marine Area | District/Local Government assisted by Ministry of Marine Affairs & Fisheries (MMAF) | 3.18 |
| 52 | Malino | Taman Wisata Alam | Nature Recreation Park | BBKSDA Sulawesi Selatan | 34.86 |
| 53 | Abdul Latief/ Sinjai | Taman Hutan Raya | Grand Forest Park | District Gov. of South Sulawesi Province | 7.39 |
| 54 | Komara | Taman Buru | Hunting Park | BBKSDA Sulawesi Selatan | 27.02 |
| 55 | Komara | Suaka Margasatwa | Wildlife Reserve | BBKSDA Sulawesi Selatan | 39.93 |
| 56 | Bontobahari | Taman Hutan Raya | Grand Forest Park | District Gov. of South Sulawesi Province | 34.30 |
| 57 | Pulo Pasi Gusung | Kawasan Konservasi Perairan Daerah | Locally Managed Marine Area | District/Local Government assisted by the Ministry of Marine Affairs & Fisheries (MMAF) | 24.05 |
| 58 | Pulau Kauna Kayuadi Kabupaten Selayar | Kawasan Konservasi Perairan Daerah | Locally Managed Marine Area | District/Local Government assisted by the Ministry of Marine Affairs & Fisheries (MMAF) | 0.43 |
| 59 | Kabupaten Morowali | Kawasan Konservasi Perairan Daerah | Locally Managed Marine Area | District/Local Government assisted by the Ministry of Marine Affairs & Fisheries (MMAF) | 104.26 |
| 60 | Teluk Lasolo | Taman Wisata Alam | Nature Recreation Park | BKSDA Sulawesi Tenggara | 8.26 |
| 61 | Mangolo | Taman Wisata Alam | Nature Recreation Park | BKSDA Sulawesi Tenggara | 39.40 |
| 62 | KKPD KABUPATEN KOLAKA | Suaka Perikanan | Fisheries Reserve | District/Local Government assisted by the Ministry of Marine Affairs & Fisheries (MMAF) | 3.07 |
| 63 | Kepulauan Padamarang | Taman Wisata Alam | Nature Recreation Park | BKSDA Sulawesi Tenggara | 37.60 |
| 64 | Murhum (Nipa-Nipa) | Taman Hutan Raya | Grand Forest Park | District Gov. of Southeast Sulawesi Province | 78.56 |
| 65 | Rawa Aopa Watumohai | Taman Nasional | National Park | National Park Agency of Rawa Aopa Watumohai | 1,072.14 |
| 66 | Lamedae | Cagar Alam | Nature Reserve | BKSDA Sulawesi Tenggara | 6.40 |
| 67 | Tanjung Peropa | Suaka Margasatwa | Wildlife Reserve | BKSDA Sulawesi Tenggara | 394.05 |
| 68 | KEPULAUAN KONAWE | Taman Wisata Perairan | Marine Recreation Park | District/Local Government assisted by the Ministry of Marine Affairs & Fisheries (MMAF) | 1.99 |
| 69 | Tanjung Amolengo | Suaka Margasatwa | Wildlife Reserve | BKSDA Sulawesi Tenggara | 4.93 |
| 70 | Tanjung Batikolo | Suaka Margasatwa | Wildlife Reserve | BKSDA Sulawesi Tenggara | 39.01 |
| 71 | Kabupaten Muna | Taman Wisata Perairan | Marine Recreation Park | District/Local Government assisted by the Ministry of Marine Affairs & Fisheries (MMAF) | 31.57 |
| 72 | Napabalano | Cagar Alam | Nature Reserve | BKSDA Sulawesi Tenggara | 0.11 |
| 73 | Buton Utara | Suaka Margasatwa | Wildlife Reserve | BKSDA Sulawesi Tenggara | 912.03 |
| 74 | Kakenauwe | Cagar Alam | Nature Reserve | BKSDA Sulawesi Tenggara | 8.13 |
| 75 | Lambusango | Suaka Margasatwa | Wildlife Reserve | BKSDA Sulawesi Tenggara | 276.04 |
| 76 | Selat Muna | Taman Buru | Game Reserve | Not Reported | 34.40 |
| 77 | Tirta Rimba Air Jatuh | Taman Wisata Alam | Nature Recreation Park | BKSDA Sulawesi Tenggara | 4.04 |
| 78 | Kabupaten Buton | Taman Wisata Perairan | Marine Recreation Park | District/Local Government assisted by the Ministry of Marine Affairs & Fisheries (MMAF) | 8.01 |
| 79 | Kepulauan Wakatobi | Taman Nasional | Marine National Park | National Park Agency of Wakatobi | 43.10 |

*BBKSDA (Balai Besar Konservasi Sumber Daya Alam) = Government Nature Conservation Agency of a Higher Level*

*BKSDA (Balai Konservasi Sumber Daya Alam) = Government Nature Conservation Agency*

**Table S2.** Under three main protection scenarios, conservation features were used to select representative areas for inclusion in Sulawesi's protected area (PA) network.

| **Conservation features** | **Coverage in Sulawesi (km^2^)** | **Area representation inside current PAs** | | **Source** |
| --- | --- | --- | --- | --- |
|  |  | **km^2^** | **Percentage** |  |
| 1.      **Carbon stocks*** | **46,155.9** | **8,109.4** | **18%** | Soto-Navarro et al., 2020 |
| 2.      **Karst ecosystem** | **24,256.6** | **1,681.2** | **7%** | MoEF 2019 |
| 3.      **Forest type** | | | | MoEF 2019 |
| a.      **Lowland (0–150 m)** | 13,925.8 | 1,911.8 | 14% |  |
| b.      **Low elevation hills (150–500 m)** | 18,545.9 | 2,233.6 | 12% |  |
| c.      **Medium elevation hills (500–900 m)** | 17,945.7 | 2,116.1 | 12% |  |
| d.      **Sub-montane (900–1,400 m)** | 18,219.7 | 3,553.5 | 20% |  |
| e.      **Lower montane (1,400–1,900 m)** | 16,578.3 | 3,530.9 | 21% |  |
| f.      **Montane (1,900–2,500 m)** | 7,615.1 | 1,788.1 | 23% |  |
| g.      **Tropical upper montane and subalpine (> 2,500 m)** | 850.7 | 241.3 | 28% |  |
| **Total forest cover** | **93,681.2** | **15,375.3** | **16%** |  |
| 4.      **Important species distribution (biodiversity)** | **105,474.4** | **16,023.4** | **15%** | IUCN Red List |
| 5.      **Ultramafic outcrops** | **15,362.9** | **1,804.9** | **12%** | MoEF 2019 |

* *The model's input data is not in terms of area size, but of tonnes of carbon per area. However, for comparison with other features in this table, we present the area size for the top quantile of carbon stock value.*

**Table S3.** The following are the 33 Red List species that were used to generate the threatened species conservation features.

| Scientific Name | Class | Red List Category | Systems | Realm |
| --- | --- | --- | --- | --- |
| *Limnonectes microtympanum* | AMPHIBIA | Endangered | Terrestrial\|Freshwater (=Inland waters) | Indomalayan |
| *Occidozyga tompotika* | AMPHIBIA | Critically Endangered | Terrestrial\|Freshwater (=Inland waters) | Australasian |
| *Oreophryne zimmeri* | AMPHIBIA | Endangered | Terrestrial | Indomalayan |
| *Ceyx sangirensis* | AVES | Critically Endangered | Terrestrial | Oceanian |
| *Coracornis sanghirensis* | AVES | Critically Endangered | Terrestrial | Oceanian |
| *Eutrichomyias rowleyi* | AVES | Critically Endangered | Terrestrial | Oceanian |
| *Zosterops nehrkorni* | AVES | Critically Endangered | Terrestrial | Oceanian |
| *Bubalus depressicornis* | MAMMALIA | Endangered | Terrestrial | Australasian |
| *Bubalus quarlesi* | MAMMALIA | Endangered | Terrestrial | Australasian |
| *Bunomys coelestis* | MAMMALIA | Endangered | Terrestrial | Australasian |
| *Bunomys prolatus* | MAMMALIA | Endangered | Terrestrial | Australasian |
| *Macaca maura* | MAMMALIA | Endangered | Terrestrial | Australasian |
| *Macaca nigra* | MAMMALIA | Critically Endangered | Terrestrial | Australasian |
| *Margaretamys christinae* | MAMMALIA | Endangered | Terrestrial | Australasian |
| *Maxomys wattsi* | MAMMALIA | Endangered | Terrestrial | Australasian |
| *Prosciurillus weberi* | MAMMALIA | Endangered | Terrestrial | Australasian |
| *Tarsius niemitzi* | MAMMALIA | Endangered | Terrestrial | Australasian |
| *Tarsius pelengensis* | MAMMALIA | Endangered | Terrestrial | Australasian |
| *Tarsius pumilus* | MAMMALIA | Endangered | Terrestrial | Australasian |
| *Boiga tanahjampeana* | REPTILIA | Endangered | Terrestrial | Australasian |
| *Calamaria acutirostris* | REPTILIA | Endangered | Terrestrial | Australasian |
| *Calamaria apraeocularis* | REPTILIA | Critically Endangered | Terrestrial | Australasian |
| *Calamaria longirostris* | REPTILIA | Critically Endangered | Terrestrial | Australasian |
| *Cylindrophis isolepis* | REPTILIA | Critically Endangered | Terrestrial | Australasian |
| *Cyrtodactylus tanahjampea* | REPTILIA | Endangered | Terrestrial | Australasian |
| *Cyrtodactylus wallacei* | REPTILIA | Endangered | Terrestrial | Australasian |
| *Dibamus manadotuaensis* | REPTILIA | Critically Endangered | Terrestrial | Australasian |
| *Indotestudo forstenii* | REPTILIA | Critically Endangered | Terrestrial | Australasian |
| *Leucocephalon yuwonoi* | REPTILIA | Critically Endangered | Terrestrial\|Freshwater (=Inland waters) | Australasian |
| *Sphenomorphus sarasinorum* | REPTILIA | Endangered | Terrestrial | Australasian |
| *Sphenomorphus tropidonotus* | REPTILIA | Endangered | Terrestrial | Australasian |
| *Trimeresurus fasciatus* | REPTILIA | Endangered | Terrestrial | Australasian |
| *Varanus lirungensis* | REPTILIA | Endangered | Terrestrial | Australasian |

**Table S4.** Cost feature used in all the three land management scenarios

| Cost Feature | Weight Score | Source |
| --- | --- | --- |
| 1. Land Cover |  | (Ministry of Environment and Forestry of Indonesia, 2019) |
| a)       Dryland Forest | a) 1 |  |
| b)       Secondary Dryland Forest | b) 1 |  |
| c)       Primary Mangrove Forest | c) 1 |  |
| d)       Secondary Mangrove Forest | d) 1 |  |
| e)       Swamp | e) 1 |  |
| f)        Primary Swamp Forest | f) 1 |  |
| g)       Secondary Swamp Forest | g) 1 |  |
| h)       Shrubs | h) 5 |  |
| i)        Shrub Swamp | i) 5 |  |
| j)        Savannah | j) 1 |  |
| k)       Plant Forest | k) 10 |  |
| l)         Plantation | l)  10 |  |
| m)     Dryland Farming Mixed Shrub | m)  10 |  |
| n)       Dryland Farming | n) 10 |  |
| 2. Integrated Forest Landscape | 0-10, a low score for integrated forest | WCS (https://www.forestlandscapeintegrity.com/ Grantham et al. 2020b) |

**Table S5.** Result of Boundary Length Modifier and iteration calibration for each scenario

| ***Scenario**** | **17%a** | **17%b** | **30%a** | **30%b** | **50%a** | **50%b** |
| --- | --- | --- | --- | --- | --- | --- |
| ***Protected areas included*** | Yes | No | Yes | No | Yes | No |
| ***BLM*** | 4.5 | 4.7 | 2.5 | 4.2 | 6.2 | 7.4 |
| ***Iterations (million)*** | 10 | 10 | 10 | 10 | 10 | 10 |
| ***Mean cost*** | 34,314 | 13,621 | 44,338 | 27,993 | 91,806 | 83,283 |
| ***Mean boundary length*** | 10,451 | 11,261 | 15,969 | 13,836 | 18,242 | 17,472 |

## Figures


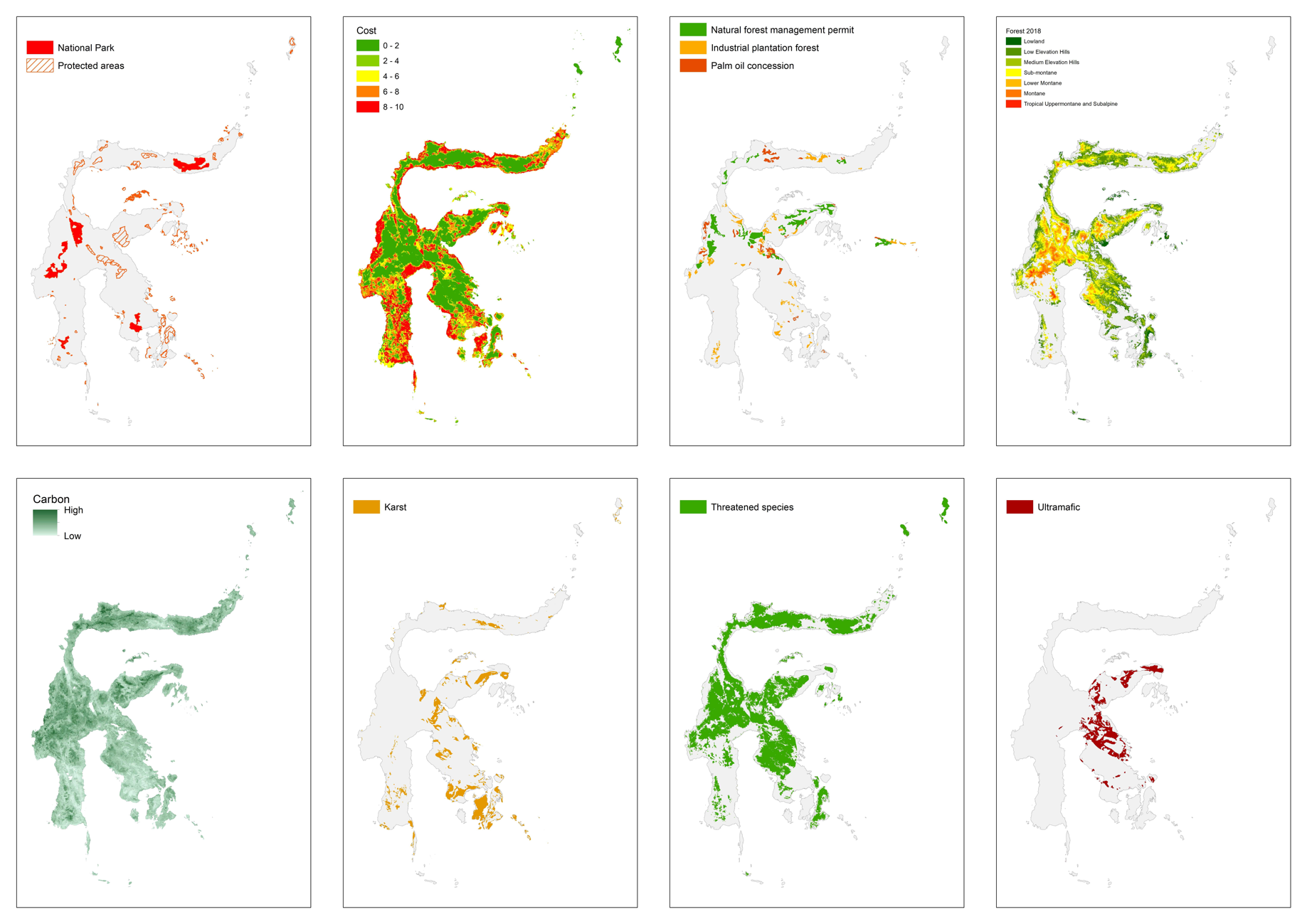


**Figure S1.** The study area with existing protected areas network (the national parks in red polygon), the potential threats from logging and plantation concessions, the cost scoring, and the three conservation features: forest cover 2018, carbon with soil organic, and karst ecosystem.

**Figure S2.** Priorities for conservation in each cluster, in order of implementation for scenario 'a'. The PA's name is the number referring to those in Table S1.


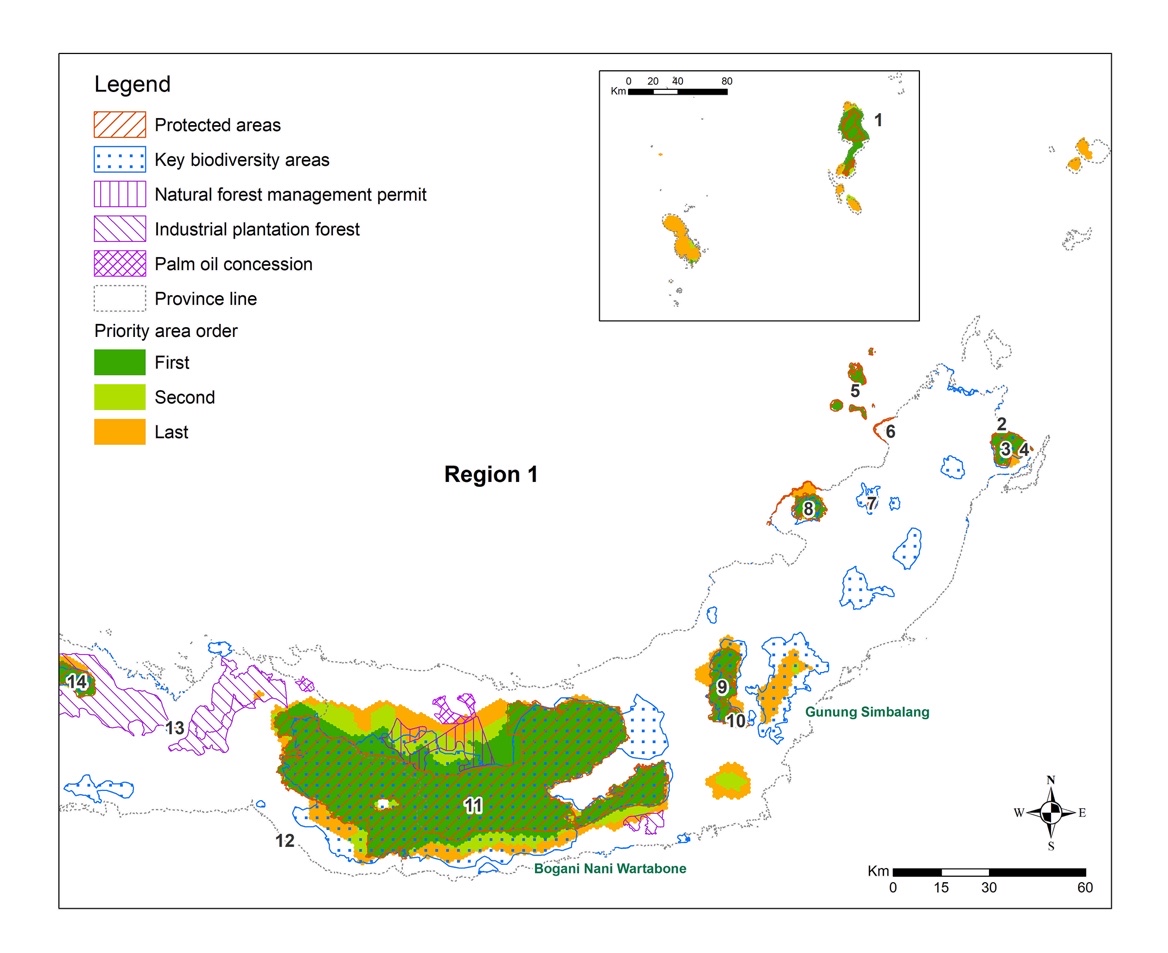

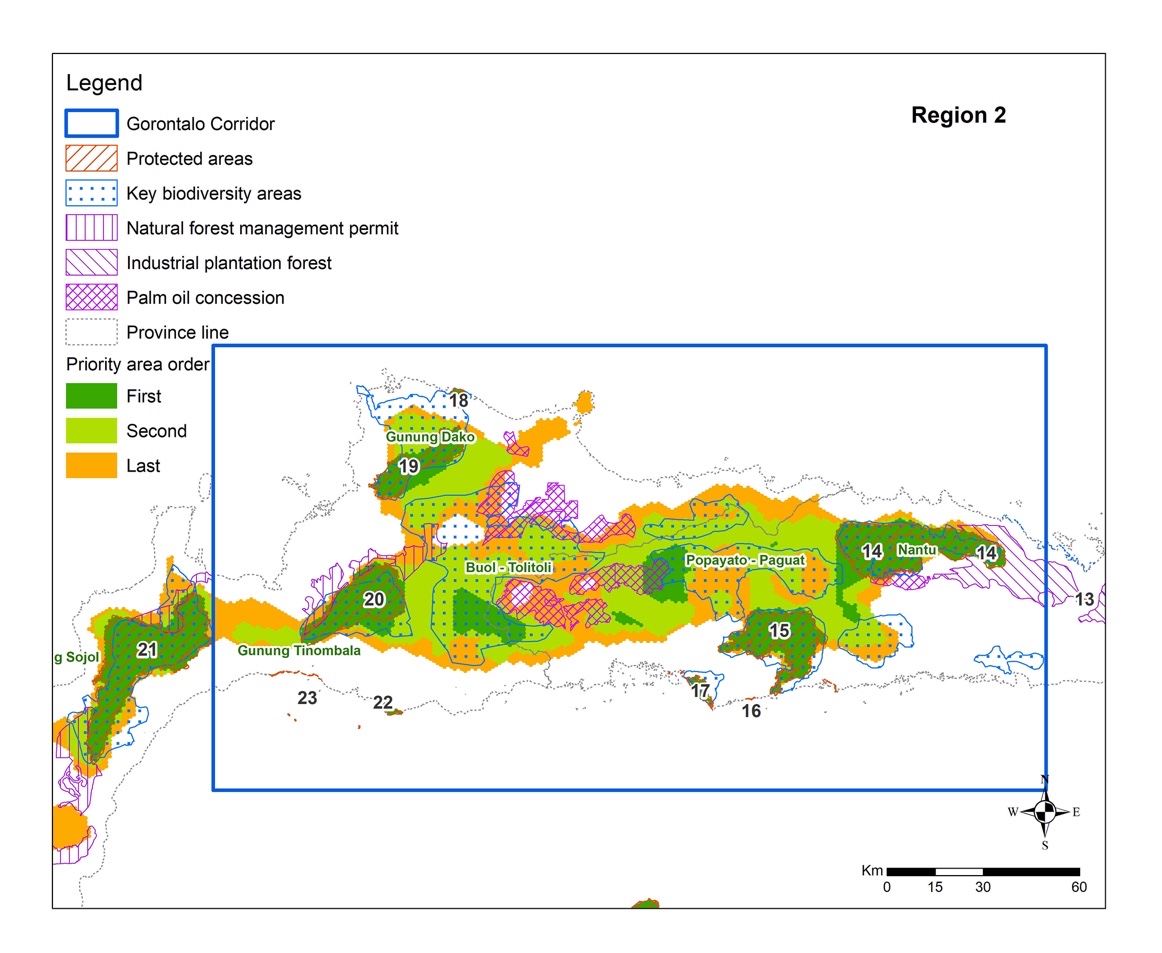

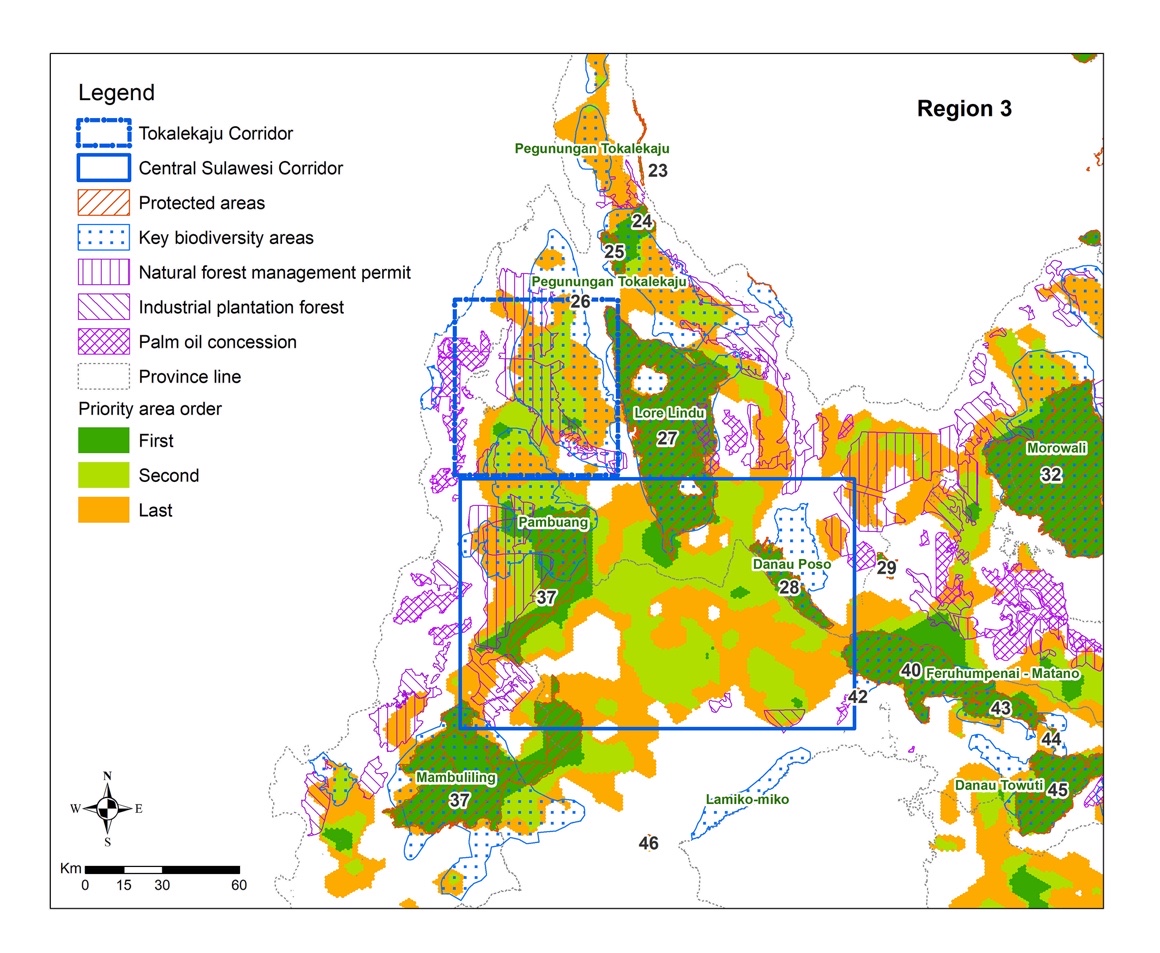

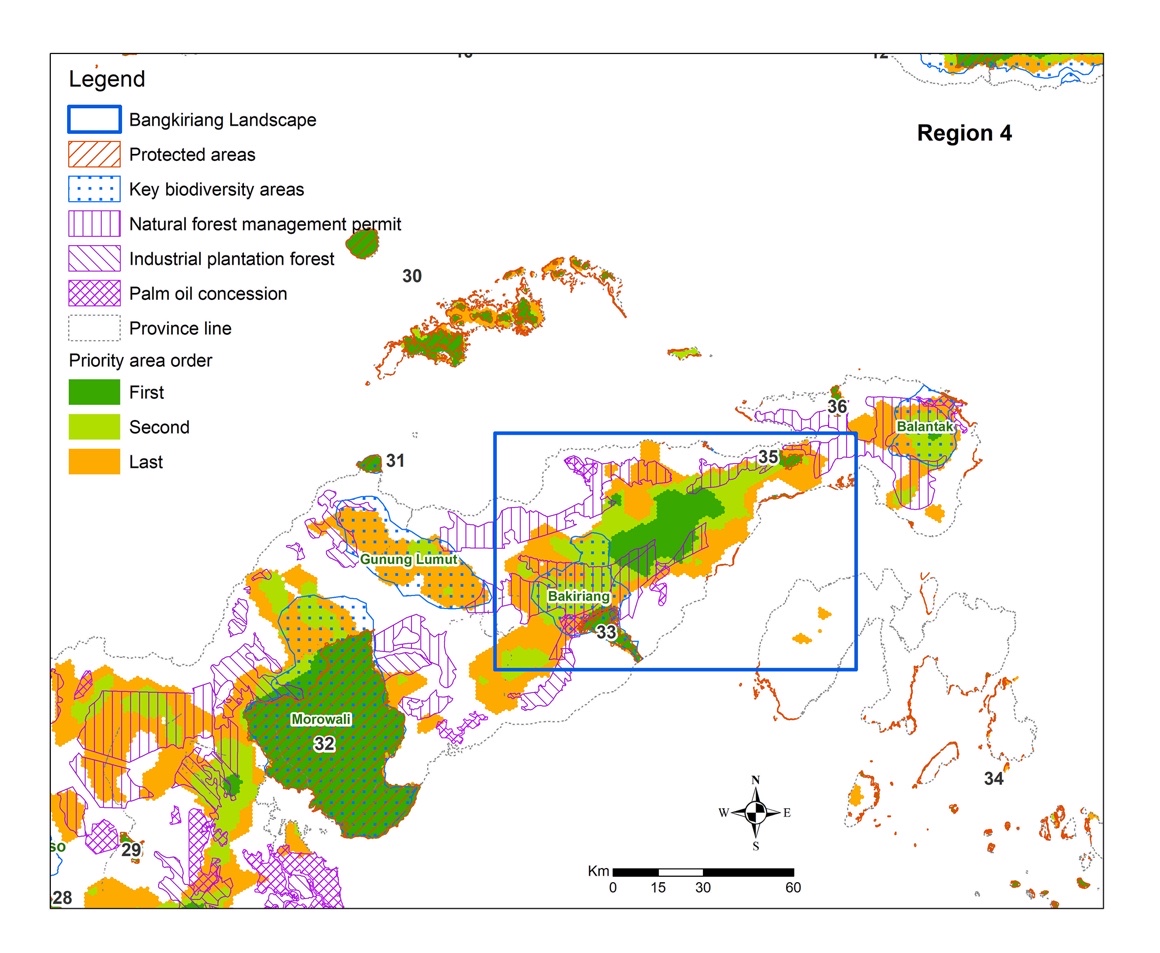

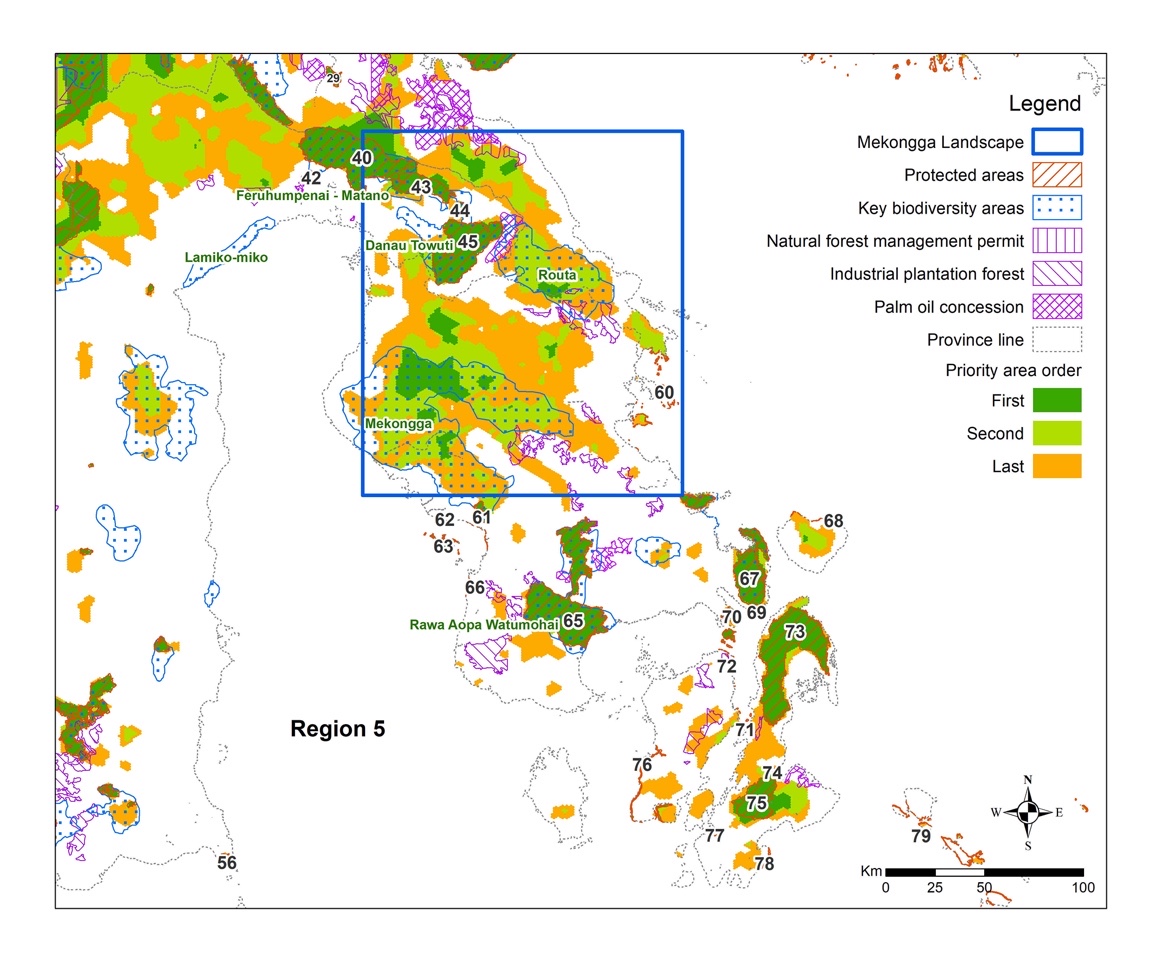

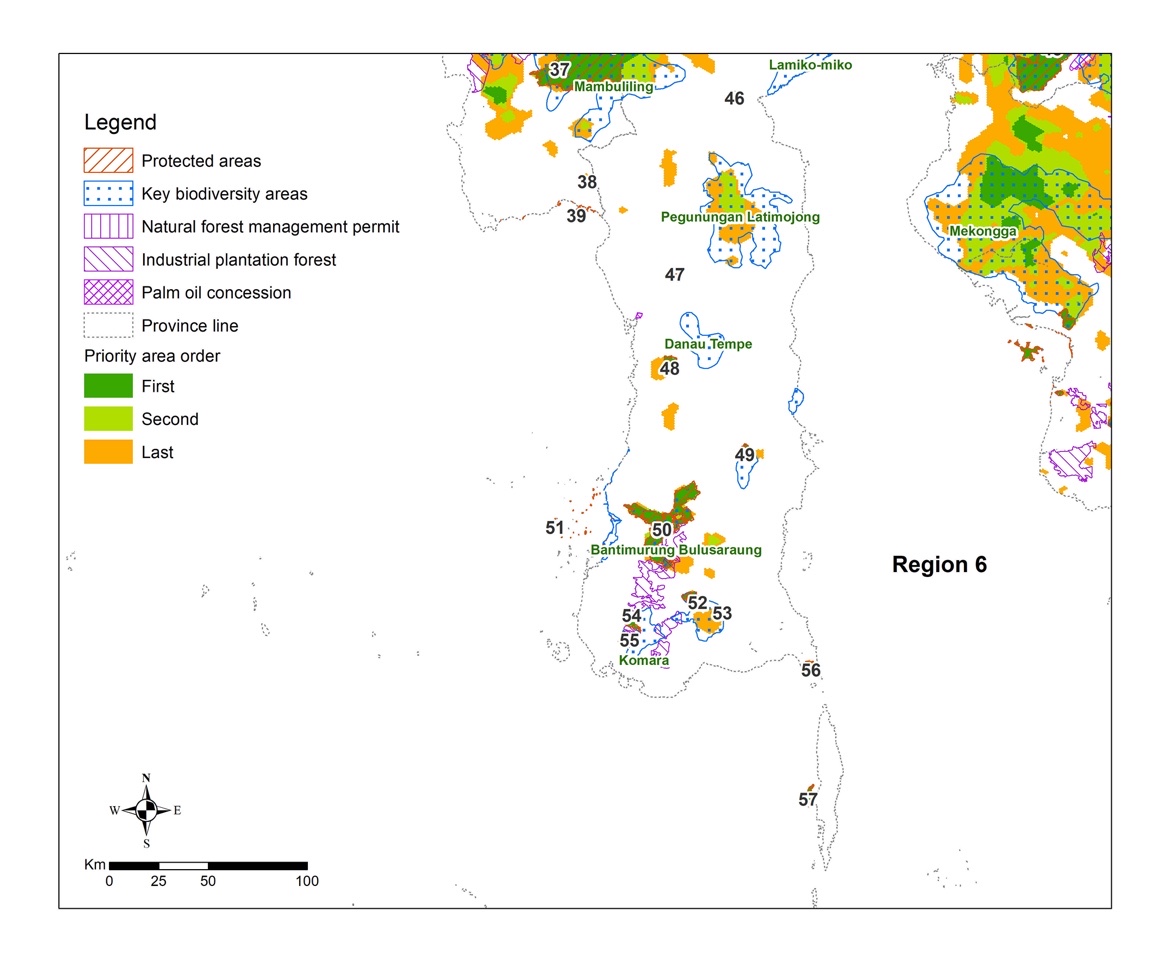


**Figure S3.** Priorities for conservation in each cluster, in order of implementation for scenario 'b'. The PA's name is the number referring to those in Table S1.


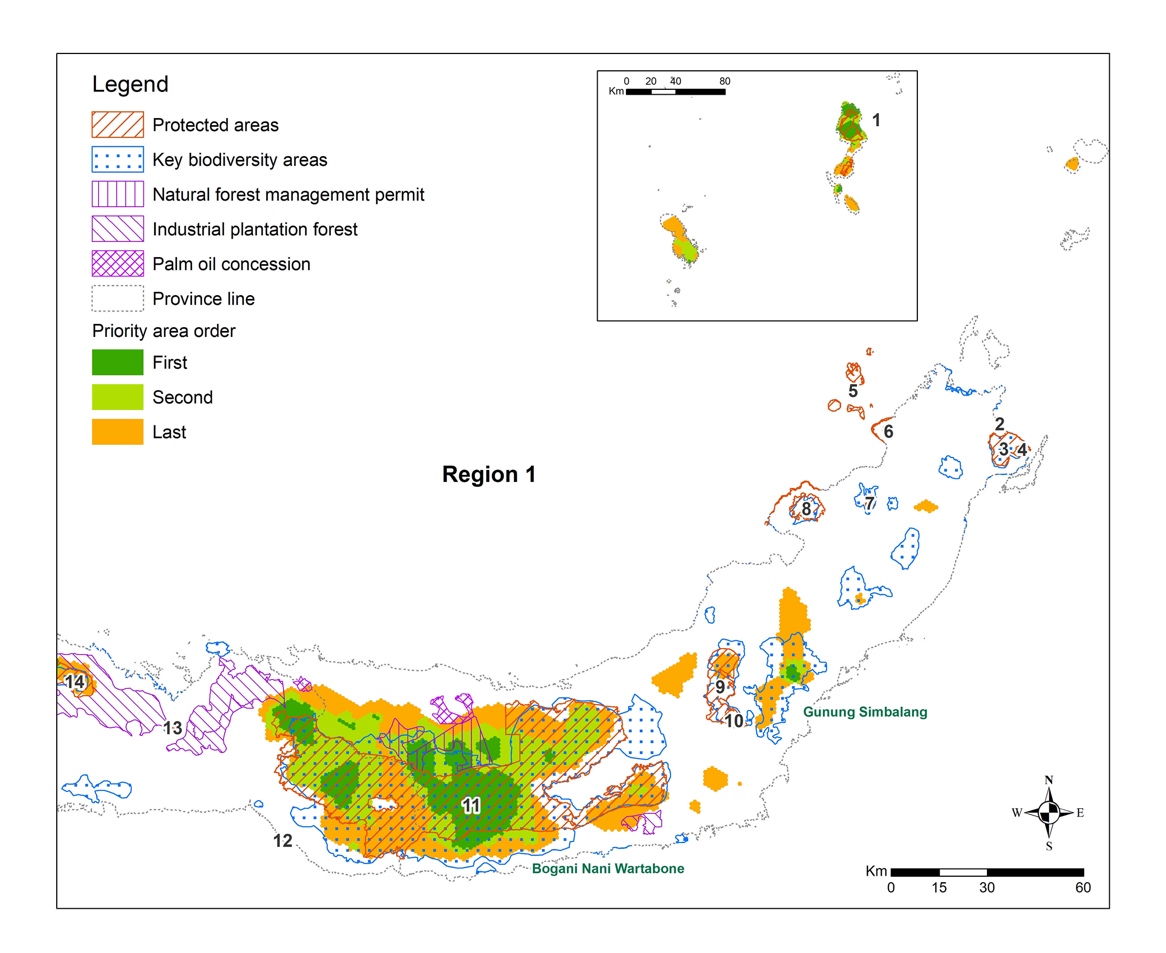

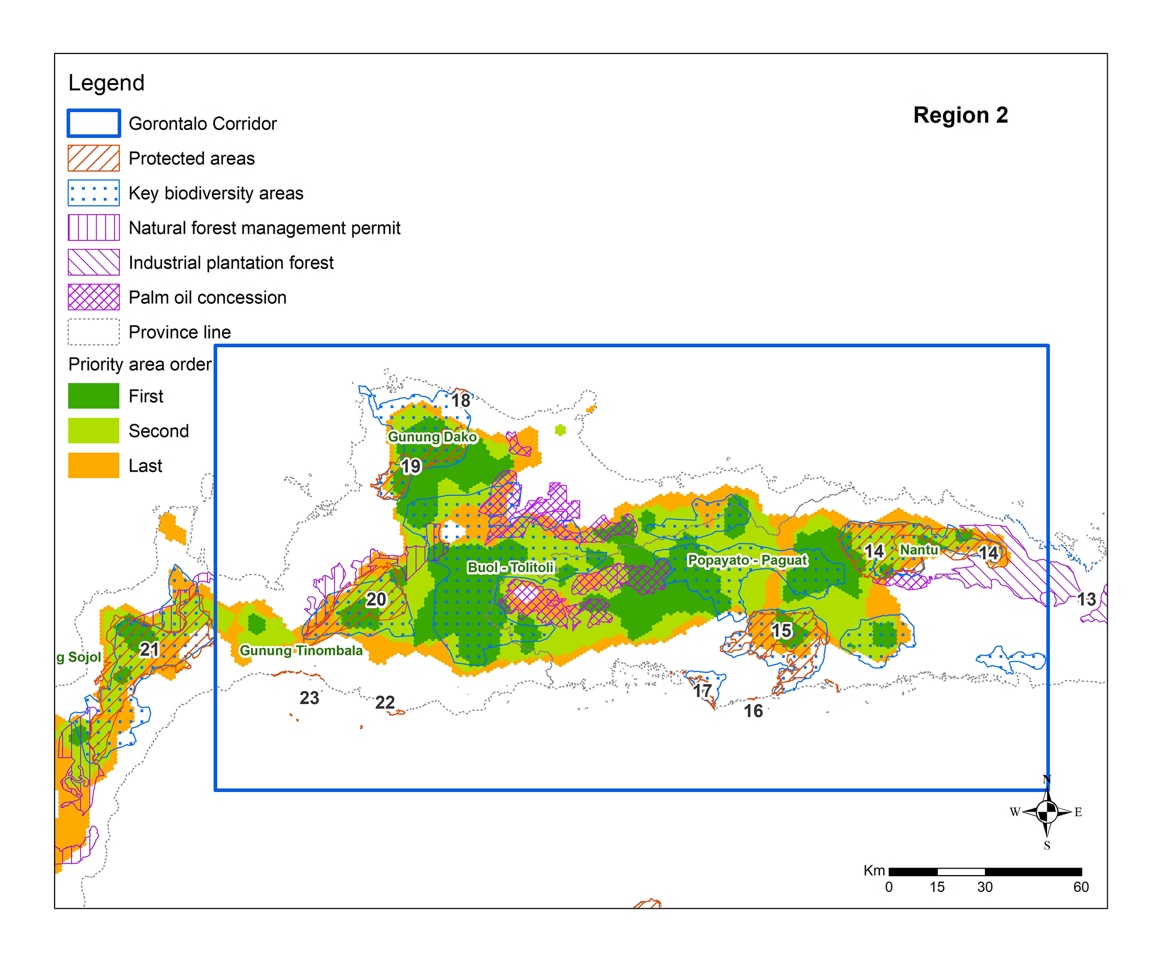

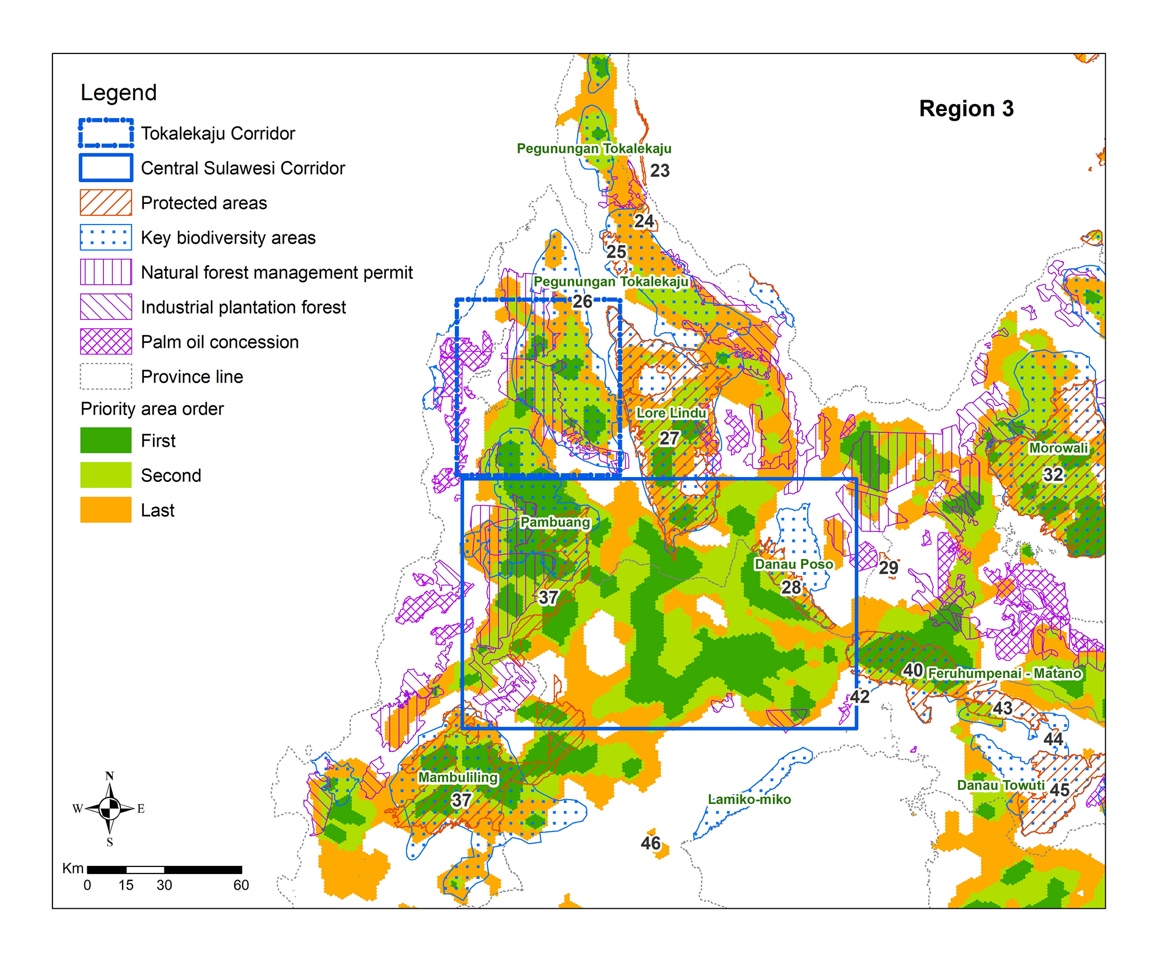

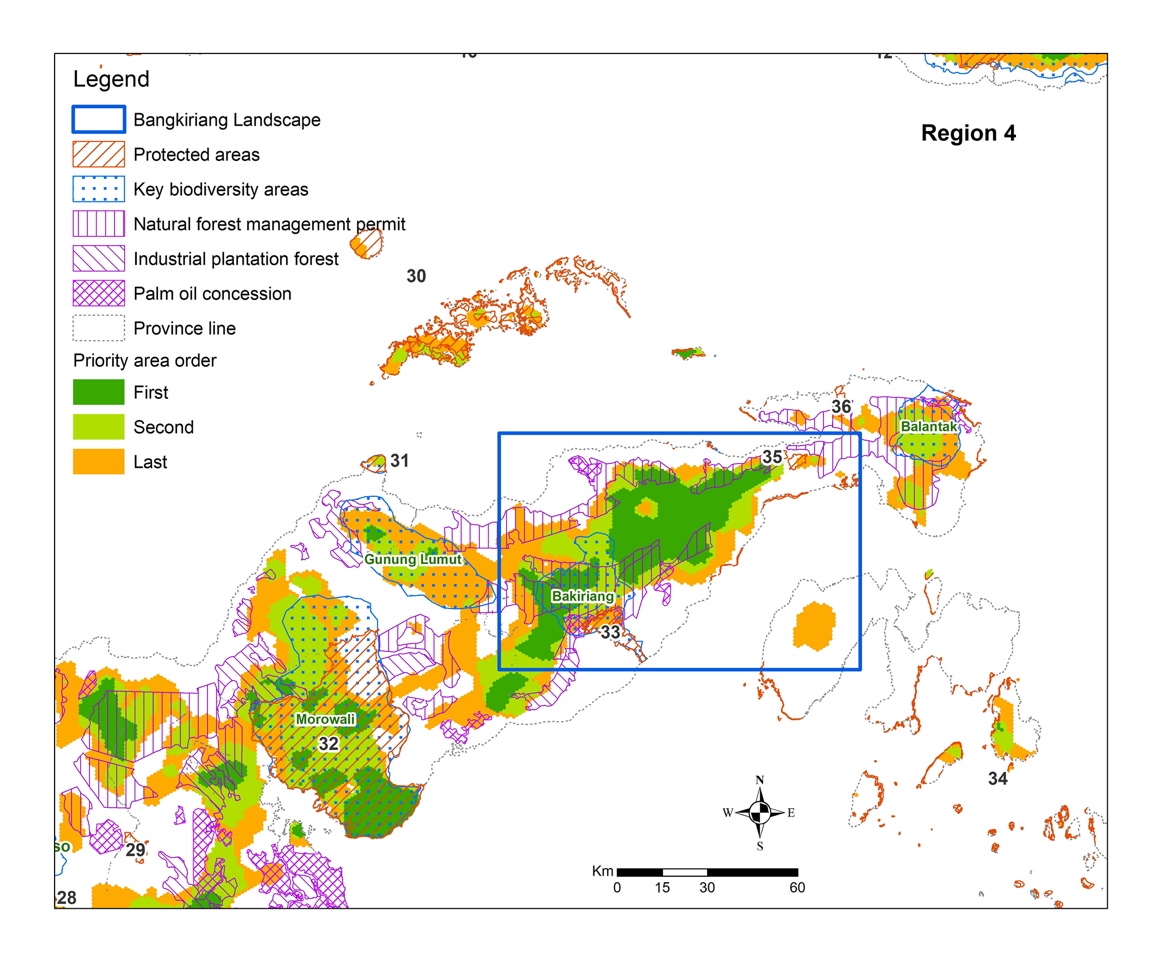

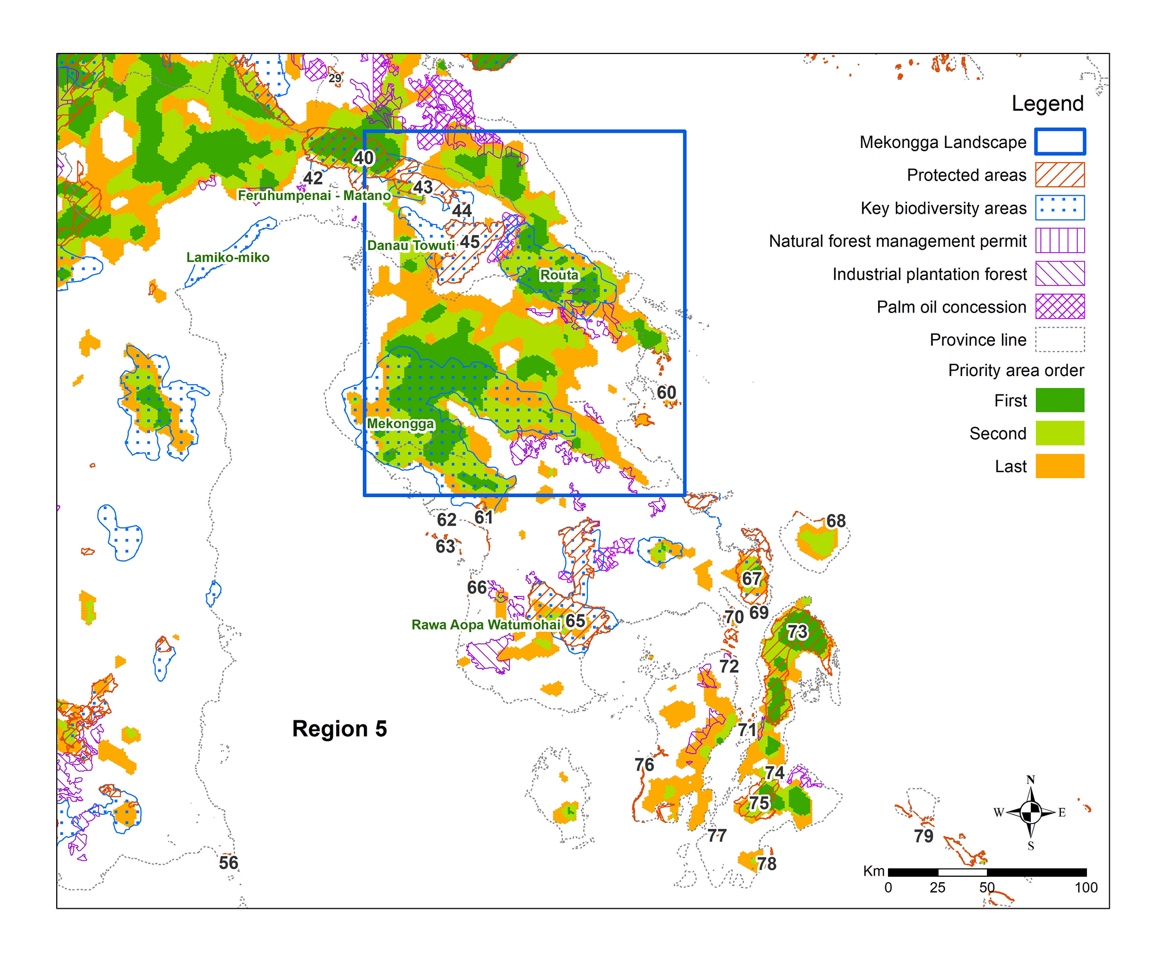

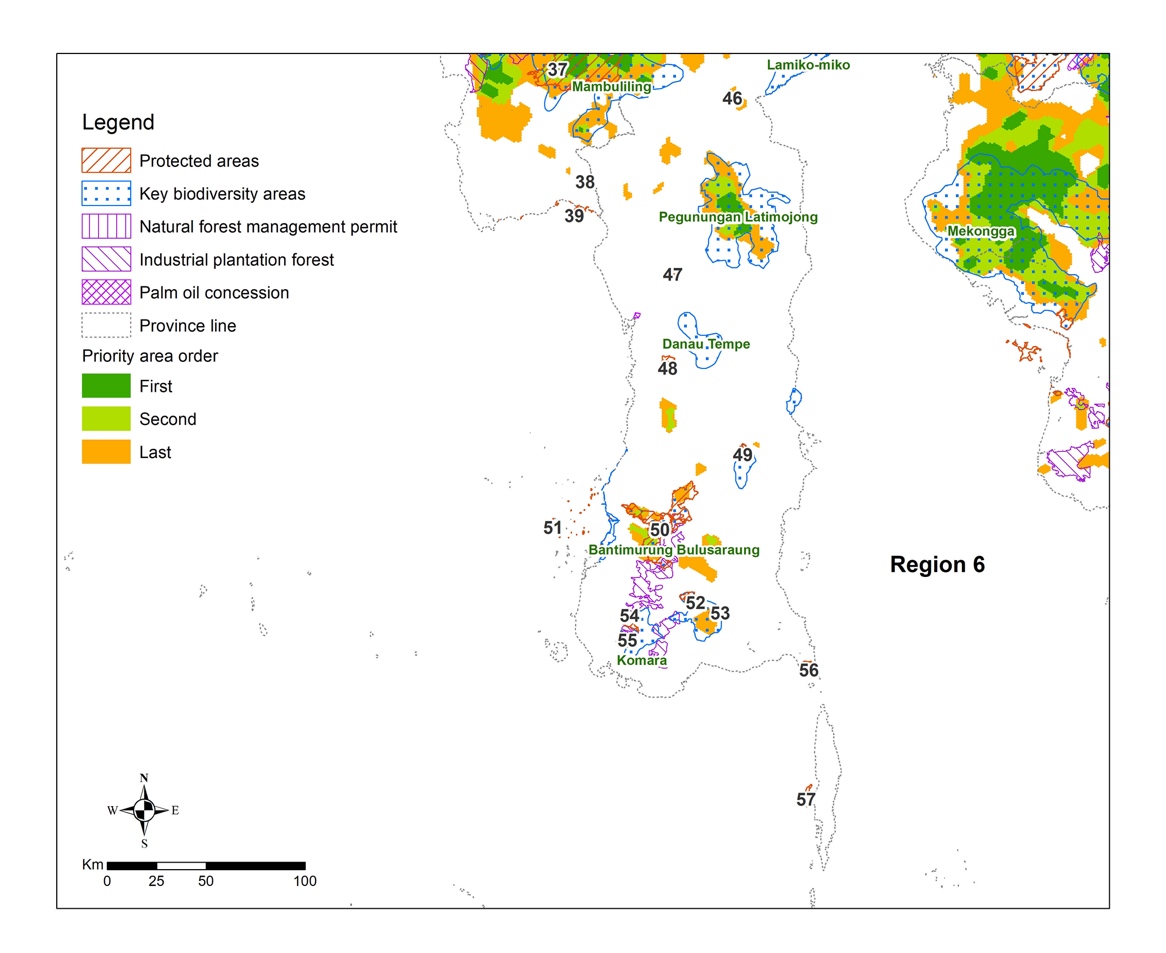


Sangihe Island
